# Barriers to Accessing Acute Care for Newly Arrived Refugees

**DOI:** 10.5811/westjem.2019.8.43129

**Published:** 2019-10-16

**Authors:** Amy J. Zeidan, Utsha G. Khatri, Michelle Munyikwa, Aba Barden, Margaret Samuels-Kalow

**Affiliations:** * Emory School of Medicine, Department of Emergency Medicine, Atlanta, Georgia; † University of Pennsylvania, Department of Emergency Medicine, Philadelphia, Pennsylvania; ‡ University of Pennsylvania Perelman School of Medicine, Philadelphia, Pennsylvania; § University of Pennsylvania Perelman School of Medicine, Department of Internal Medicine, Philadelphia, Pennsylvania; ¶ Massachusetts General Hospital, Department of Emergency Medicine, Boston, Massachusetts

## Abstract

**Introduction:**

Over the past decade, the number of refugees arriving in the United States (U.S.) has increased dramatically. Refugees arrive with unmet health needs and may face barriers when seeking care. However, little is known about how refugees perceive and access care when acutely ill. The goal of this study was to understand barriers to access of acute care by newly arrived refugees, and identify potential improvements from refugees and resettlement agencies.

**Methods:**

This was an in-depth, qualitative interview study of refugees and employees from refugee resettlement and post-resettlement agencies in a city in the Northeast U.S. Interviews were audiotaped, transcribed, and coded independently by two investigators. Interviews were conducted until thematic saturation was reached. We analyzed transcripts using a modified grounded theory approach.

**Results:**

Interviews were completed with 16 refugees and 12 employees from refugee resettlement/post-resettlement agencies. Participants reported several barriers to accessing acute care including challenges understanding the U.S. healthcare system, difficulty scheduling timely outpatient acute care visits, significant language barriers in all acute care settings, and confusion over the intricacies of health insurance. The novelty and complexity of the U.S. healthcare system drives refugees to resettlement agencies for assistance. Resettlement agency employees express concern with directing refugees to appropriate levels of care and report challenges obtaining timely access to sick visits. While receiving emergency department (ED) care, refugees experience communication barriers due to limitations in consistent interpretation services.

**Conclusion:**

Refugees face multiple barriers when accessing acute care. Interventions in the ED, outpatient settings, and in resettlement agencies, have the potential to reduce barriers to care. Examples could include interpretation services that allow for clinic phone scheduling and easier access to interpreter services within the ED. Additionally, extending the Refugee Medical Assistance program may limit gaps in insurance coverage and avoid insurance-related barriers to seeking care.

## INTRODUCTION

Over three million refugees have been resettled in the United States since Congress passed the Refugee Act of 1980.^1^ In 2015, there were nearly 70,000 new refugee arrivals, representing 69 different countries.^1^ Refugees undergo pre-departure health screening prior to arrival in the U.S., and are typically seen by a physician for an evaluation shortly after arrival.^2^ Refugees are resettled in areas with designated resettlement agencies that assist them with time-limited cash assistance, enrollment in temporary health coverage, and employment options. Refugees are initially granted six to eight months of dedicated Refugee Medical Assistance, which is roughly equivalent to services provided by a state’s Medicaid program.^3^ Following this period, refugees are subject to the standard eligibility requirements of Medicaid.^3^

It is important to highlight the differences between a refugee, an asylum seeker and a migrant, as this study focuses specifically on refugees. A refugee is an individual who has been forced to leave his or her home country due to fear of persecution based on race, religion, nationality, membership in a social group, or policital opinion. Refugees undergo robust background checks and screening prior to receiving designated refugee status. They are relocated only after undergoing this screening process, and have legal protection under the Refugee Act of 1980 given their status as a refugee. An asylum seeker, on the other hand, is an individual who has fled his or her home country for similar reasons but has not received legal recognition prior to arrival in the U.S. and may only be granted legal recognition if the asylum claim is reviewed and granted. As a result, asylum seekers do not have access to services such as Refugee Medical Assistance, time-limited cash assistance, or similar employment opportunities. Migrant is a general term and refers to an individual who has left his or her home country for a variety of reasons.^4^,^5^

Prior studies have shown differences in utilization of the emergency department (ED) by refugees in comparison to native-born individuals.^6^ In Australia, refugees from non-English speaking countries are more likely to use ambulance services, have longer lengths of stay in the ED, and are less likely to be admitted to the hospital.^6^ A study conducted in the U.S. evaluated refugees one year post-resettlement and demonstrated that language, communication, and acculturation barriers continue to negatively affect their ability to obtain care. These data suggest that there may be unidentified opportunities for improving the acute care process for refugee populations; however, little is known about how refugees interface with acute care facilities. Therefore, the goal of this study was to use in-depth qualitative interviews to understand barriers to access of acute care by newly arrived refugees, and identify potential improvements from refugees and community resettlement agencies.

## METHODS

### Study Design

Because the healthcare experience of refugees has not been well described and they cannot be reliably identified in administrative datasets,^7^ we chose to conduct an in-depth interview study to identify the potential barriers and facilitators to accessing acute care as a newly arrived refugee. We included the following in the definition of acute care: sick visits, urgent appointments with the patient’s primary care doctor, urgent follow-up with specialists and dentists, urgent care visits, and ED visits. Because our goal was to understand the range of experiences rather than the number of times an experience is identified, we chose in-depth interviews to obtain a detailed understanding of the perspective of each respondent. Interviews were conducted until thematic saturation was reached, when the data no longer identified new perspectives or themes.^8^ We used purposive sampling to balance across gender to ensure that the fullest range of perspectives was included.^9^

**Population Health Research Capsule**

What do we already know about this issue?

*There is limited data describing refugee use of and barriers to acute care services in the U.S.; however, studies outside the U.S. suggest that barriers exist*.

What was the research question?

What barriers do newly arrived refugees face when accessing acute care in the U.S.?

What was the major finding of the study?

*Refugees face multiple barriers when accessing acute care, but interventions in and outside the emergency department may reduce barriers to care*.

How does this improve population health?

*Understanding the barriers that refugees face and working with resettlement agencies to reduce barriers may improve their health status and health outcomes*.

### Study Setting and Population

We conducted the study at a refugee clinic and at resettlement and post-resettlement agencies. The refugee clinic was located at a tertiary care hospital in a city in the Northeast U.S. The clinic has been in operation for approximately five years and has cared for approximately 200 refugee patients yearly. At the time of the study, the clinic received referrals from one of the three resettlement agencies in the city. Refugee patients were seen within 30 days of arrival. Most refugees were seen for screening evaluations and transitioned to clinics near their homes after two to three clinic visits. Refugee patients were eligible for this study if they were over 18 years of age, had capacity to consent, and had no hearing difficulties. We excluded refugees if they were deaf, unable to answer questions from an interpreter, or had acute medical or psychiatric illnesses.

In the city in which the study was performed, there are three main resettlement agencies and approximately three well-known post-resettlement agencies. Resettlement agencies are responsible for receiving new refugee arrivals and assisting individuals with support for three to six months after arrival. Resettlement employees assist refugees with establishing housing, employment, transportation, primary care, and language services. After three to six months, refugees are able to seek additional assistance at post-resettlement agencies. Post-resettlement agencies provide additional support in terms of support groups, language services, cultural activities, and case management. Employees were eligible for this study if they worked at a resettlement or post-resettlement agency, were over 18 years of age, and had no hearing difficulties.

### Study Protocol

This was an in-depth interview study using semi-structured, open-ended interviews. Separate interview guides for refugees and resettlement agency employers were developed by all members of the study team. Study team members included the following: an emergency physician and investigator with expertise in qualitative methodology (MSK); an internal medicine physician with many years of experience working at the refugee clinic (AB); a third-year emergency medicine (EM) resident with three years of experience working bimonthly at the refugee clinic (AJZ); a second-year EM resident with no experience at the refugee clinic (UGK), an MD/PhD student with three years of experience working at the refugee clinic and content expert on refugee studies (MM); and an undergraduate student with two years of experience working at the refugee clinic (EJ). The study team composition allowed for a range of expertise with individuals who had experience working with refugees and those who did not. Questions were vetted among the all members of the study team and revised to ensure that content reflected the goals of the study. Prior to interviewing resettlement and post-resettlement employees, a resettlement/post-resettlement employee interview guide was developed using the same process. (See Appendix A for interview guides.)

Refugee interviews were conducted in person at a refugee clinic, and refugees were recruited during the study period when an interviewer was present during clinic hours. Refugees were asked to participate if a room and interpreter were available. If the aforementioned conditions were met, all refugees awaiting clinic appointments or available after their appointment were asked to participate. All of the refugees who were asked agreed to consent and participated. Interviews with refugees were conducted by two members of the study team (AJZ and EJ) using the Refugee Interview Guide (Appendix A) and lasted approximately 30 minutes. A phone interpreter was used for verbal consent prior to participation and for the interview. Demographic information was collected about each participant (see Appendix A). After interviews were completed for refugee patients, a second phase of semi-structured, open-ended, interviews were conducted in person at local resettlement and post-resettlement agencies in the region.

We obtained a list of employees involved in case management, health coordination, and program development for refugees/immigrants from resettlement healthcare teams. These employees were contacted via email with information regarding the study and consent form. Of 13 employees contacted, 12 participated. Employee interviews were conducted at their respective agencies, and verbal consent was obtained prior to participation. Interviews with resettlement employees were conducted by two members of the study team (AJZ and MM) using the Resettlement/Post-resettlement Employee Interview Guide (Appendix A) and lasted approximately 20 minutes. This study was approved by the institutional review board at the University of Pennsylvania.

### Data Analysis

Each interview was recorded, professionally transcribed, and coded by three investigators (AJZ, EJ and UGK coded refugee interviews, and AJZ, UGK and MM coded resettlement interviews). The study team met regularly to design and refine a coding scheme for the refugee interviews. A separate coding scheme was developed for interviews with resettlement/post-resettlement agencies and similarly was refined regularly. All coding differences were resolved by consensus. (See Appendix A for codebook.) Interviews were conducted until consensus on thematic saturation was reached. The study team defined thematic saturation as the point when information obtained in interviews no longer revealed new information regarding barriers faced by refugees when accessing acute care.

## RESULTS

### Demographics

A total of 16 interviews were completed (12 men, 4 women) with refugees. Participants had a mean age of 34 (range 20–48) and 50% had completed high school. Countries of origin were Syria (5), Bhutan (2), Democratic Republic of the Congo (2), Burma (2), Sudan (2), Iraq (1), Iran (1) and the Central African Republic (1). Most refugees seen at this refugee clinic undergo medical screening within one to two months of arrival. A few of the patients remained at the clinic for long-term follow-up. All refugees required an interpreter and all interpretation was done with phone interpreters. A total of 12 interviews were completed for resettlement and post-resettlement agencies. Resettlement employees interviewed represented two resettlement agencies and two post-resettlement agencies.

**Figure 1:**
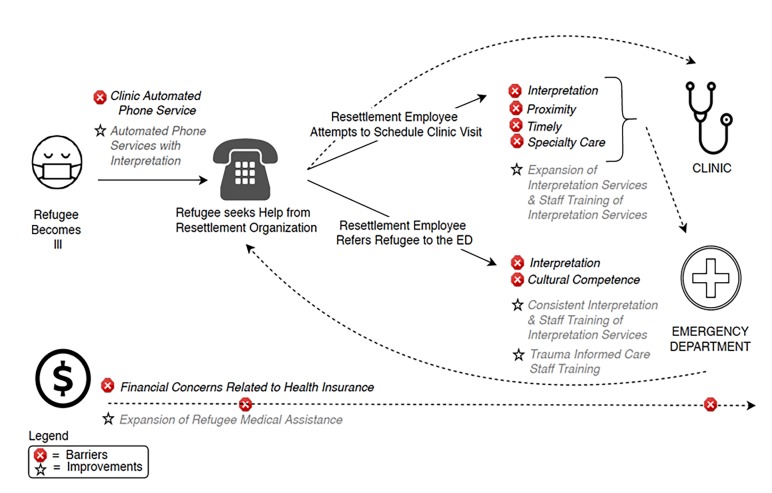
Process of seeking acute care for newly arrived refugees: barriers and potential solutions.

We identified several barriers to access of acute care facilities by newly arrived refugees (Table 1). The process by which refugees seek care and barriers at each step can be visualized in Figure 1.

### Pre-Acute Care Expectations

Prior to seeking care, refugees are influenced by their past experience with health systems, which vary considerably depending upon the country/countries where they lived previously. The ED is often a new healthcare setting for refugees that differs significantly from those in their country of birth or origin. Additionally, many refugees report that they are unaware of hospitals or clinics close to their house but do know how to call 911.

“They [refugees] are not aware where they should go [when sick]. One of the clients had a high fever. So they ran to the emergency department. They’re not used to the United States healthcare system because in their culture they just go to the hospital, which might not be just for emergencies.” (Resettlement Employee)

“I do not know [where to go if I’m sick] because I’m new here in the United States. I only know one thing. If my condition worsens a lot, then I can just dial 911.” (Refugee)

### Reliance on Resettlement Agencies

The uncertainty and unfamiliarity of the new healthcare system drives many refugees to the resettlement agency. Refugees reported relying on resettlement agencies for their needs and healthcare information. Refugees reported seeking advice from resettlement agencies prior to seeking care, often treating the resettlement agency as a triage center. Resettlement agency employees often have non-medical backgrounds and reported concern when providing medical advice to refugees, often referring to the ED depending upon the perceived severity of illness.

“We refer refugees to the ER all the time. We do not have licensed medical staff who are able to diagnose and treat patients in the office. So if they come in with anything life threatening or if they come in with something that they feel is an emergent issue, chest pain or something along those lines, we refer to the emergency department.” (Resettlement Employee)

### Barriers to Acute Outpatient Care

For those refugees who attempt to schedule outpatient care when sick, they experience significant difficulty with scheduling sick visits due to availability of same-day or next-day visits and language barriers with automated telephone services. As a result, they often rely on resettlement agencies for scheduling sick visits. Resettlement agency employees reported frequently scheduling appointments for patients because of language barriers. However, both resettlement agency employees and refugees reported that obtaining timely appointments for sick visits was challenging.

“I called to get an appointment for my son. He was not feeling good. He has asthma. And they told me they didn’t have an appointment until June [many months away]. I had to take him at 3:00 in the morning to the hospital.” (Refugee)

Aside from difficulties with scheduling, other common challenges included finding primary care clinics that accept Refugee Medical Assistance, offer interpreter services, and are geographically convenient. Resettlement agency employees also reported significant difficulty in finding specialists, mental health providers, and dentists who care for refugees, as they often do not accept Refugee Medical Assistance and may have less-robust interpretation services available. Notably, resettlement agency employees commented more on these challenges than refugees. Resettlement agency employees reported scheduling appointments for refugees regularly due to language barriers.

“But mostly, for example, in the northeast part of the city or somewhere else with private or small clinics they are not familiar with the interpretation services or they rely on family members, even kids, to help them to interpret which is really – I always advise my clients not to depend on that. And even the parents, sometimes they don’t feel comfortable sharing their medical concerns with their kids [as interpreters].” (Resettlement Employee)

### Barriers in the ED

When refugees do seek care in the ED, they report challenges obtaining interpretation throughout the entire ED process and limited explanation of the process including timeline and results.

“I went with one woman [to the ER] who spoke French because she wanted someone to accompany her. And the ER was expecting me to be the interpreter. I was like you know, you guys need to call an interpreter. I’m not trained to do this.” (Resettlement Employee)

Resettlement employees reported the desire for more culturally competent care in the ED, specifically citing trauma-informed care. Resettlement employees felt that refugees may present with somatic complaints resulting from their history of torture, trauma, and the stress of resettlement. These symptoms can be difficult to triage, diagnose, and treat both for resettlement employees and for medical teams alike.

“I see a lot of people saying they have a heart problem. And when you ask them – were you diagnosed with a heart problem before? They say no but I feel my heart is beating out of control. I’m not a healthcare provider, but it seems like a panic attack or anxiety.” (Resettlement Employee)

### Complexity of Health Insurance

Finally, refugees and resettlement employees reported confusion regarding the concept and complexity of health insurance, a barrier that is present at each point of access in the healthcare system. Most countries where refugees were born or lived prior to arrival do not have health insurance or have systems that differ significantly from the insurance structure in the U.S. Resettlement employees observed that refugees have many misperceptions of the insurance system and were often overwhelmed about paying for medical care and insurance.

“I get tons of bills from emergency departments because the clients either didn’t know to give them the [insurance] card or they thought they were uninsured – assumed they were uninsured.” (Resettlement Employee)

When refugees were asked if they knew what health insurance was, the responses were varied:

“Life insurance?”

“Something for free? Provides meds and treatment that the state provides to the people.”

“A paper from the hospital?”

“Eight months of coverage, could be extended, but will eventually expire.”

“Covers fees for getting sick, gives access to a doctor. Necessary to make preventative appointments.”

“Something that lasts for eight months, then I have to pay out of pocket which will be very, very expensive.”

### Recommendations from Resettlement Employees

A majority of resettlement employees suggested interventions to reduce barriers and improve how refugees interface with the healthcare system. Outside of the ED, recommendations largely focused on improving access and resources for dedicated outpatient care and providers for refugees. For ED providers, resettlement employees stressed the importance of using trained interpreters and educating providers on how to provide culturally competent care. They also recommended educating refugees on appropriate ED utilization (Table 2).

## DISCUSSION

Our principal findings identify barriers throughout the process of accessing acute care for newly arrived refugees. Overall, refugees face uncertainty when accessing acute care services because of prior experiences in their home countries and limited understanding of the complex U.S. healthcare system. The unfamiliarity with the U.S. healthcare system drives refugees to rely heavily on resettlement employees as an initial point of triage or, if they are very sick, to call 911. At the resettlement agency, employees express concern about identifying the appropriate level of care to which to send a refugee client. They report challenges obtaining timely access to sick visits with primary care doctors and urgent visits with specialists and dentists.

Additional barriers that make obtaining unscheduled care challenging include identifying clinics that offer comprehensive interpretation services, accept Refugee Medical Assistance, and are geographically convenient. Scheduling appointments over the phone, specifically automated services, is particularly challenging for refugees with limited English proficiency. On arrival to the ED, the same language barriers create challenges to understanding care received. In addition, the lack of trauma-informed care can hinder the appropriate workup and treatment of symptoms. Finally, after obtaining care in any acute care setting, refugees face significant financial risk due to limited understanding of the health insurance system.

It is important to highlight that some of the aforementioned barriers to acute outpatient care reported exist among U.S.-born individuals, including geographical and insurance barriers, and difficulty accessing mental and dental services. However, these challenges are exacerbated for refugees due to language and cultural barriers. The U.S. healthcare system is new and often quite different from health systems refugees have used in the past, adding an extra layer of complexity to understand. The lack of interpretation services limits already limited resources such as appointments with specialists, dentists, and mental health providers. Additionally, refugees have unique mental healthcare needs given their history of trauma that adds an additional challenge when identifying appropriate mental health services.

There is limited existing data on the utilization of acute care services by refugees in the U. S. In Australia a study evaluating the use of emergency services by refugees suggested that some refugees know how to call for emergency help, yet have significant fear of calling for help because of security implications faced previously in their home countries.^10^ In our study, refugees identified knowing how to call 911 if they were ill but did not express fear as a barrier to using this service. It is possible that the study population perceived less fear because the resettlement employees recommended the use of 911.

A qualitative study in the U.S. evaluating healthcare barriers of refugees one year post resettlement also identified individual and structural barriers to accessing health services. Barriers included challenges with language, acculturation processes, and cultural beliefs.^11^ Similarly, our study found that language and acculturation were significant barriers when accessing health services. Our study differed in that we were specifically focusing on barriers to acute care access and that we identified additional barriers related to health insurance and perceived poor access to prompt outpatient clinic options. Additionally, our results identified the important role of resettlement agencies in addressing these barriers. Notably, our study occurred early in the resettlement process, a time when resettlement agencies are typically more involved, as opposed to one year after resettlement.

Respondents identified several areas for improvement to reduce barriers to accessing care for newly arrived refugees (Figure 1). Areas for improvement within the acute care system include establishing partnerships with resettlement/post-resettlement agencies to assist with triage of refugees with acute conditions, and developing specific protocols that may help resettlement employees direct patients to appropriate levels of care. Finally, respondents recommended incorporating cultural competency and trauma-informed care training for providers. Trauma-informed care is based on the premise that past exposure to trauma can have long-lasting effects on the physical and mental health of patients. Thus, providers and organizations can respond by adopting trauma-informed models of care.

A trauma-informed organization acknowledges that trauma is pervasive, recognizes the signs and symptoms of trauma, and integrates knowledge about trauma into policies, procedures and practices with the goal of avoiding retraumatization.^12^ While it is challenging to accurately estimate the number of refugees who have experienced trauma prior to resettlement, estimates suggest that the prevalence rate may be as high as 35%.^13^,^14^ This does not account for trauma associated with the resettlement process. ED-specific approaches of trauma-informed care have been suggested for violently injured patients who have been injured due to violence and are treated in the ED; and some components may be applicable to refugee populations.^15^ While more research is needed to establish trauma-informed models of care for refugees in the ED, providers should acknowledge a patient’s history of trauma, ongoing signs and symptoms, and avoid practices that may result in retraumatization.

A major theme in our interviews was the importance of interpretation services. Refugees and resettlement employees describe challenges at all points of acute care access due to language barriers and a lack of appropriate interpretation services. Revisions to the Affordable Care Act in 2016 mandated that healthcare facilities must offer qualified interpreters to limited English proficient (LEP) patients^16^ and the 2010 Joint Commission standards also require qualified interpreter services in hospital settings.^17^ However, patients with LEP have worse clinical outcomes and receive a lower quality of care.^18^ In the ED formal interpretation should be offered to all patients who do not identify English as their primary language, and operation teams should ensure interpretation services are embedded throughout a refugee’s ED course, and that all members of the ED team are routinely trained on how to use in-person and phone interpreters. Similarly, clinic teams can ensure that interpretation services are available during clinic visits, but also when refugees call to schedule appointments or ask questions.

Another common barrier reported by resettlement employees and refugees is that refugees struggle to understand health insurance, which is also supported in prior studies.^19^ More education for refugees was suggested as a potential intervention to address this concern, and may be useful. However, additional policy changes may be required to avoid insurance-related barriers to accessing care. For example, refugees who live in states without Medicaid expansion have a much smaller chance of enrolling in health insurance once Refugee Medical Assistance ceases.^20^ Additionally, it has been reported that in states where Medicaid requires reapplication annually, refugees often have a gap in insurance coverage.^19^

A study evaluating health coverage for immigrants suggests that expanding universal coverage may actually reduce net costs for LEP patients by increasing access to primary prevention and reducing emergency care for preventable conditions.^21^ For refugees, the cessation of Refugee Medical Assistance after eight months occurs at a difficult time of transition. At six to eight months, cash assistance from the government typically ends as does support from the resettlement agency based on the expectation that refugees are self-sufficient after six to eight months of support.^2^,^3^ A study evaluating unmet needs of refugees demonstrated that refugees in the U.S. for a longer period of time are more likely to report a lack of health insurance coverage and a delay in seeing a healthcare provider.^22^ Policymakers should consider extending Refugee Medical Assistance beyond the first eight months as an additional strategy to improve access to health insurance and ensure stable access to care.

Finally, additional research is needed to understand networks of care for refugees. In order to understand ED utilization by refugees and barriers to acute care, future studies should focus on prospectively following refugees after arrival to identify patterns of use and integration long term. This would then help guide types of interventions at locations where refugees most frequently seek acute care. Systematic identification of refugees in national datasets would assist with understanding variations in patterns of utilization between different regions and identifying areas of particular importance.

## LIMITATIONS

We obtained the data from this study from one city. This limits the generalizability as results may be specific to the refugee experience in this location and healthcare system. However, our sample engaged refugees from a variety of countries, representing the current distribution of refugees resettled to locations throughout the country. This study did not specifically evaluate differences in access to acute care barriers for refugees based on country of origin, gender, educational, cultural, or economic background; however, all of these factors may influence experiences and are important to consider in future studies. Interviews with refugees occurred at a refugee clinic affiliated with a local resettlement agency and did not include refugees without acces to care and services. Similarly, resettlement agency employees were recruited by the study team, largely consisting of physicians.

Interviews with refugees were conducted mostly within three months of their arrival, thus only targeting newly arrived refugees. Barriers to access may differ at different stages of the resettlement process. However, this early period is likely to be the most vulnerable time with significant language, acculturation, and financial challenges. In addition, refugees typically see a physician within 30 days of arrival in the U.S. Many resettlement agencies work with specific clinics to meet this goal, making this the optimal time to capture a diverse population receiving care at one location.

Some members of the study team had significant experience working at the refugee clinic and may have been influenced by potential biases from previous work with refugees, specifically when identifying themes. To counter these potential biases, members of the study team included individuals who did not work at the refugee clinic. Transcripts were double coded by both a clinic and non-clinic investigator and reviewed by a non-clinic investigator.

Additionally, the use of interpreters may have altered responses from refugee patients. In some languages, a direct translation for specific words or meanings may not exist and as a result may be translated in a meaning that is different than what was intended. Finally, as with all qualitative studies, results generate hypotheses from the experience of the participants rather than testing or measuring a hypothesis.

## CONCLUSION

Our data demonstrate that there are multiple barriers refugees face when accessing acute care. Participants described barriers to timely outpatient care and significant challenges accessing ED care and understanding the complexities of health insurance. These results offer patient and stakeholder data to support implementation and evaluation of novel interventions focused on expansion of insurance coverage, enhanced access to quality interpretation, and targeted research efforts that will improve care provided to refugees.

## Supplementary Information



## Figures and Tables

**Table 1:** Themes & Illustrative Quotes.

Theme	Illustrative quotes
Pre-acute care expectations	**[Resettlement Employee]** “At the beginning they will be confused between can we call 911 in these situation because they are used to in their countries to go – to show up doctor office any time and without an appointment and the doctor will see them.” **[Refugee]** “I think the only place to go is the hospital when I get sick or one of my family get sick. Because I don’t know doctors. I don’t know private clinics.” **[Refugee]** “But it’s very difficult to get medication, because in [my home country] it’s a very different way to get medication. You can just go to the pharmacy and you can get any medication. But here, it must be a prescription.” **[Resettlement Employee]** “I receive many questions about the prescription, how can we fill it. How can we go to the pharmacist and ask them to. This is a challenging thing.”
Reliance on resettlement agencies	**[Refugee]** “If I became sick, I’ll still go back to them [resettlement employee] and ask them for help, because they are the only ones that I know. So I’ll still go and ask them how I can go about it and how I can manage to see a doctor.” **[Resettlement Employee]** “If I get a call and someone says, I can’t breathe – and it could be their tonsils are swollen and it’s hard for them to breathe, but because I’m not a medical professional, and when I get that call, I have to kinda – I talk it through, but the safest thing for me is to say, yes, go [to the ED].”
Barriers to acute outpatient care	**[Resettlement Employee]** “For non-native English speakers, that is an increased barrier because they don’t know how to get through the automated phone system.” **[Resettlement Employee]** “I think specifically for follow-up visits, I feel like it’s a little on the slower side. I feel like some of our clients, it takes over a week sometimes, just because the clinics are so busy.” **[Resettlement Employee]** “We’ve had several issues when – seeing the dentist, they need deep cleaning. It’s not covered by insurance. It’s like $200.00. So they can’t afford that.” **[Resettlement Employee]** “There’s just such a shortage of mental health care providers that are either covered by insurance or who are able to accommodate for non-English speaking patients.”
Barriers in the ED	**[Refugee]** “I felt that my sugar level was down, so I went to the dentist […] they examined my sugar level, they referred me to the hospital. They did some bloodwork for me. But they did not tell me about the results. I would like to know about the results at least.” **[Resettlement Employee]** “I have a 60-year-old client; I think she’s having panic attacks, going to the ER. I took her one time […] when they said they’re going to discharge at midnight but didn’t provide transportation. A 60-year-old, no language, where she’s gonna go? So she was told to sit in a lobby until in the morning to go home. Next time, I asked them, I said, what’s the plan of discharge? Is she gonna have transportation or an ambulance taking her back? I wanna know if she’s gonna get a taxi.” **[Resettlement Employee]** “The idea of navigating the sort of westernized healthcare System […] people think oh, I just have to go the emergency room and they’ll sit there for hours. They’re gonna give me a pill. I’m gonna get this huge bill. And it’s just gonna mask the pain.”
Health insurance barriers	**[Resettlement Employee]** “I don’t blame them because in their countries, they don’t have the health insurance. Sometimes you don’t need it because it’s free health system.” **[Resettlement Employee]** “I had an incident where a woman was having a miscarriage and experiencing heavy bleeding. And she was calling me and another coworker at 10:00 at night. She had been at work and didn’t know what to do because she was experiencing this heavy bleeding, but didn’t want to leave work early because she was afraid about losing her job and she didn’t think that her Medicaid would pay for the ambulance ride. But that’s an example of people just having misconceptions about how their health insurance works and how the system works.”

*ED*, emergency department.

**Table 2:** Recommendations from Resettlement Employees.

Location	Recommendation
Outpatient	At dedicated refugee clinics, increase availability and timeliness of appointments, dedicate specific times weekly for refugee appointments, ensure consistency of medical providers and provide one central number patients and resettlement employees can call when medical questions arise. Develop a paid community health worker certification program to provide care navigation to refugees including accompaniment to the pharmacy, medical appointments, and for assistance with health insurance questions. Include and train social workers in this process if available. Provide basic medical training for refugee resettlement employees so they can better assist with triaging patients.
ED	Provide cultural competency training to providers to improve comfort with taking care of populations with different cultural backgrounds and implementing dedicated training on use of both in person and phone interpreters. Educate patients on the process of going to the pharmacy and filling prescriptions as pharmacies do not have interpreters. Develop a protocol for refugees regarding when to go the ED and educate refugees on how to use the protocol.

*ED*, emergency department.
