# Supplementary material for: Barriers to Accessing Acute Care for Newly Arrived Refugees
Source: West J Emerg Med. 2019 Oct 16;20(6):842–50. doi: 10.5811/westjem.2019.8.43129 (PMC6860387; doi:10.5811/westjem.2019.8.43129)
Supplement: Supplementary file 1 [file wjem-20-842-s001.docx]

**Refugee Interviews Brief Demographic Survey**

Demographic questions for refugees:

1. What is your age?
2. What country did your parents come from?
   1. What country were you born in?
   2. What country did you live in before coming to the US?
3. What is your gender?
4. Did you go to school?
   1. If so, what year of school did you finish?
5. What languages do you speak?

*For interviewer only:

1. What kind of interpreter was used for this interview ?

**Resettlement/Post-resettlement Agency Employee Interview Guide**

This interview guide was developed by the study team and refined used during interviews with resettlement and post resettlement agency employees.

| Initial Question | Follow up question 1 | Follow up question 2 |
| --- | --- | --- |
| Can you tell me about your role at the resettlement organization? |  |  |
| What are the biggest challenges you face in your position? |  |  |
| What are things you find rewarding in your position? |  |  |
| Is education provided to refugees through your organization about health care in the US? | If so, describe | When is this provided? |
| Do refugees discuss their health concerns or health status with you? | Can you describe what they talk to you about?  What are the most **common** health concerns they ask you about? | Do they ask you specific health related questions? If so, what kinds of questions do they ask you? |
| Do refugees ask you about health insurance? If so, what questions do they ask? | Do you think they understand what health insurance is?  If no, why do you think this is? | Where do you think refugees get information about health insurance, if not from your organization? |
| Do you feel refugees have adequate access to health care? | What are challenges you feel refugees face with regards to their health? | What are challenges you feel refugees face with regards to health care? |
| What do you think would help improve the health care we (as a medical system) provide to refugees? | What solutions would you recommend be considered to improve the health care provided to refugees through your organization? | What solutions would you recommend be considered at clinics/health centers to improve the health care provided to refugees? |
| Have you ever referred a refugee to the Emergency Department | If yes, what was it for? | If yes, did you talk to them after the fact? If yes, what was their experience like? |
| Have you ever referred a refugee to the clinic for a sick visit? | If so, what was it for? | Did you talk to them after the fact? If yes, what was their experience like? |
| What additional training/support (for health providers or you) would help you in your role support refugees with their health concerns? |  |  |

**Refugee Interview Guide**

This interview guide was developed by the study team and refined during interviews with refugees.

| Initial Question | Follow up question 1 | Follow up question 2 |
| --- | --- | --- |
| Tell me about your health. How would you describe your health? | Do you think you are healthy? | How do you feel about your health? |
| What helps you stay healthy? | What are things that help you take care of your health? |  |
| What makes it difficult to take care of your health? |  |  |

| Who/what helps you take care of your health? | Is there anyone in your life who helps you? |  |
| --- | --- | --- |
| Whose advice do you trust for taking care of your health? | Is there someone you ask when you have questions about your health |  |
| If you have questions about your health, how do you learn about your health or where to go to learn about your health questions? | How do you like to learn information about your health? Internet? Books? Individuals? |  |

| When you were in X country (where you moved from), where would you go when you became sick? | How would they help you? |  |
| --- | --- | --- |
| Now, in the US, where would you go if you become sick? |  |  |
| Did you receive information about health care services/resources when you arrived? If so, what? | What did the agency tell you about healthcare resources? | What information do you wish you had about healthcare in the United States? |
| 1Do you know what health insurance is? If yes, what is it? |  |  |

| Did you talk to anyone in X country about health care in the US before you came to the US? If so, what did they say? | What are things about health care in the US that are surprising to you and that you were not expecting prior to moving to the US? |  |
| --- | --- | --- |

| What do you wish your health care providers knew about your health? Is there anything you wish your doctor knew about your health? | What do you wish they knew about you? |  |
| --- | --- | --- |

| What was your experience moving to the US like? | What were your first impressions about the United States | What did you think when you first arrived. How did you feel? |
| --- | --- | --- |
| What made your experience as a refugee harder? | What are challenges or difficulties you faced |  |
| What made your experience as a refugee easier? |  |  |

| What did you wish you knew before arriving to the US |  |  |
| --- | --- | --- |
| Do you have any questions? |  |  |

**Codebook for Resettlement/Post-resettlement Agency Employee Interviews**

This codebook was developed by the study team to code the interviews with resettlement and post-resettlement agency employees. The study team met regularly to design and refine the codes below. Codes are listed in bold with descriptions of the code following.

**Descriptions of Codes**

1. **Level of care:**
   1. This includes comments about where a person chooses/has chosen to obtain care including comparisons regarding where to obtain care.
2. **Language:**
   1. Includes comments about language barriers and interpretation.
3. **Knowledge & Perception of Health Insurance:**
   1. This category includes comments about health insurance
4. **Knowledge & Perception of US health care (different systems):**
   1. This category describes comments about the US healthcare system
5. **Access:**
   1. Includes comments pertaining to the act of or ability to access care. This can include barriers (physical distance from clinic/hospital, transportation, automated phone servers that make access difficult, etc).
6. **Non-health care services:**
   1. This category includes anything non-health care related; example, employment, school, etc.
7. **Ideas for improvement:**
   1. Recommendations for improving the care process for refugees
8. **Relationship with agency:**
   1. Comments about resettlement and post-resettlement employees/programs and their assistance/relationship with patients.

**Codebook for Refugee Interviews**

This codebook was developed by the study team to code the refugee interviews. The study team met regularly to design and refine the codes below. Codes are listed in bold with descriptions of the code following.

**Descriptions of Codes**

1. **Non healthcare resources and experiences**
   1. These are experiences other than healthcare experiences that patients report. For example, experiences in general, experiences with food instability/stability, employment, housing, transportation, general finances.
   2. This category is divided into those experiences other than healthcare experiences that happened prior to arriving in the US and those that occurred after arrival. Any comparison of their pre-US experience to a US experience is coded in the post category.
2. **Values/priorities/emotions/family**
   1. This is a category to capture emotions, priorities and values that do not fall under non health care ‘experiences’. It focuses on the emotional aspect or value behind the statement. If patient’s discuss an experience, this would fit in category 1. If they describe an emotion/value, it would fall in this category. Often times people talk about their family or children when referring to emotions of values, which falls in this category as well.
3. **Health Status and Perception**
   1. This describes the way someone feels about and perceives their health. How they describe their health; it can include their perception of their health with regards to how others make them feel about their health
   2. This category is divided into pre US and post US to understand how patient’s perceived their health prior to coming to the US, and how they perceive their health after arrival. This will help us understand if there’s a difference, and evaluate if their perception of their health or their actual health status changes. For any comparison of health status pre US vs post US, it should fall under the post US category.
4. **Knowledge, understanding and perception of the US Health Care System**
   1. This category refers to statements in which patients answer questions and discuss their understanding of health care in the United States and about how the health system works (reality or perceived). It’s divided into 4 categories
      1. Barriers: Things that prevent patients from navigating and understanding the health system
      2. Facilitators: Things that help patients navigate and understand the health system
      3. Informal Social Network: Individuals that patients recognize as people who help them navigate and understand the health system
      4. Provider relationship: How patients perceive and reflect on the relationship they have with their doctor
5. **Access to Healthcare**
   1. This category is separate from category 4 in that it captures they physical component of going to a health care facility. It extends beyond simply knowledge of going to a health care facility but actually knowing about one and/or where it is located. It extends beyond the theoretical understanding to the realistic understanding of a health care facility.
   2. This category is divided into pre US and post US to understand the actual access patients had to health care prior to arrival and now in the US.

| CODE | SUBCODE | QUOTE |
| --- | --- | --- |
| Nonhealthcare resources and experiences |  |  |
| Nonhealthcare resources and experiences | Pre US | Q: Can you tell me why you describe your health as medium health  A: Well because I didn’t live a normal life like everyone else. I didn’t grow up in a normal environment. I had to work starting from the morning and returning back home in the night. I didn’t have a childhood. So that’s why. (3_49)  Q: how did you feel when you learned you were coming to the United States?  A: Yes. We were living a very unstable life in Jordan, been there for six years. And our life was just a mess, completely a mess. We were very [inaudible 00:03:57] very uncomfortable whatsoever. And my kids were not in school the right way. My husband even found a job, but didn’t find out that he found a job, they would have to deport us back. The place we were staying was not comfortable, was not safe either. So it was just a mess (4_51)  Q: So how did you feel when you learned you were coming here?  A: Yes, ma’am. I did feel tense a little bit, I did. You coming to a place you don’t know nothing about, you don’t know the culture, you don’t know nothing. So I was a little tense about that (4_51)  Q: Did you talk to anyone, prior to moving, about the United States?  A: My mother told me that, tried to come earlier, when you come here, your problems will be solved, you will have a good life over here. You don’t have to worry about anything else and you will enjoy a better life over here. She told me that. That is all (6_53)  Q: Is there anything that you wish you knew before coming to the United States?  A: So actually, no, my answer is no. I just [inaudible 00:33:08] when I came here to the states, I only had probably $70-90 dollars. And before [inaudible 00:33:17] brought me here to the states, I studied American history. I didn’t know a country that has only two to three hundred years, only, was able to reach – I mean go to the moon and have all that great history, that other countries who have been there for like 2000 years wouldn’t be able to do any of that. And also, I think – I wasn’t able to do anything before I came to the states, because in Syria, I lost everything. I lost my job, I lost my business, I lost my car. And back in Egypt, I was like zero, I had zero money. So I don’t think [inaudible 00:34:10] I’ve been here now in the US (7_54)  Q: Did you talk to anyone about the healthcare in the United States before you moved here?  A: Nobody told me, because the kind of life that I had back home, I mean that I had back home, in my situation and when I was in Kenya I didn’t have anyone to talk to, I didn’t even have a place to stay. Sometimes I would sleep on the ground (9_55)  Q: What was your experience like moving to the United States?  A: I was so happy. I was so happy about it, because I had the very, very bad life in Kenya. I was sleeping outside. [inaudible 00:10:38] would help me. But then my life was very, very hard at the time. So when I – when they told me I was coming, I was excited, because I thought now I’d have a place to stay, I’d have food to eat, I wouldn’t be hungry (9_55) |
| Nonhealthcare resources and experiences | Post US | Q: How did you feel when you first got here? (1_47 line 17)  A: Yeah, I was very happy, mainly because there were no more stumbling blocks about my employment issues. (1_47 line 18-19)  Q: what has made your experience harder? (1_47 line 20)  A: The first thing I experienced was – back there it was like a week or two of any time you want it to, so – but when I came here, it was – the weather was very cold, and there was kind of a little bit restricted about leaving the house – restriction about leaving the house because of the weather (1_47 line 21-24)  Q: What has made your experience as a refugee in America easier? (1_47 line 27)  A: back there, there were restrictions, even about buying some – for example, a phone. You would have to have paperwork. Plus, it was one rule for the natives and one for the refugees, like us. So once I got here, and I have all the legal paperwork, I can – and do whatever I want, and I have documented – documentation to show my identity. So things are much easier (1_47 line 28-32)  Q: what did you wish you knew before coming to the US? (1_47 line 33-34)  A: My only regret is that I did not go to school properly. I did not – I should have finished school or attended more – like say, taking more care of my education. Because the first thing – first of the hurdle – the biggest stumbling block here is my lack of the English (1_47 line 35-37)  Q: How do you plan to go about learning English? (1_47 line 38)  A: I need to attend classes and be more aggressive with my English learning, but then again, there’s still – I have to fulfill my job obligations as well. So that’s – on one hand, there’s a need for – to learn English, and on the other hand is a need to earn money by getting a job (1_47 line 39-42)  Q: Were there any things that made your experience, coming to the United States, more difficult? (2_48)  A: The only thing was, still is, language barrier, not to be able to communicate with people (2_48)  Q: Are there any things that have made your experience easier (2_48)  A: There was nothing actually, accept for the language barrier actually. Other things weren’t different actually from the place I came from (2_48)  Q: Was there anything that you wish you knew before coming to the United States (2_48_  A: I wish I could bring my qualifications actually with me, because I didn’t know that, I thought okay there would be a test, for continuing education. But now I’m here and I see that okay, they do need my previous background, educational background for me (2_48)  Q: Are there any other things, besides your kids, that would make it difficult to go see a doctor, for example, cost or transportation or any other things that would make it difficult?  A: So yes, of course, these are kind of difficulties for me. But I’m not dependent on the money and everything in my life. I just need a little bit of money just to keep me living normally and independently, and that’s okay. I don’t need that much (3_49)  Q: Is there anything in the US that’s different than what you expected  A: Well there is too many things that I didn’t expect to happen, like the insurance for me and my wife, the agency will help us paying for our expenses for the first three months. There is the food card that is also a kind of benefit. We are buying food with it. And also the cash assistance. So I didn’t know that they exist. (3_49)  Q: can you just tell me a little bit more about your experience when you first arrived in the United States?  A: So it was a very good experience. The people that received us, they were so kind. I didn’t – I am from Syria and it doesn’t matter from where I am. So I just wanted to tell you that here, we saw kindness and we saw respect for all the system, that is very beautiful. And the system is over all the people and it’s over everything. And we have to respect everyone. Everyone here is respected. Although he has another religion, another race, or something, everyone is free to say anything. And we are really happy to be here and to be free and democracy (3_49)  Q: Was there anything that made your experience in the US more difficult?  A: No. There was no difficulty, per say, but my condition is difficult, because I have to be in the hospital and be in the house and be at school. So just that (3_49)  Q: And what were your first impressions about the United States, once you got here?  A: The first thing I felt when I got here to United States is that I’m a human again and I’m normal and I’m living again. It’s not like when I was Jordan. Everybody use to just point at you, like a refugee from Syria. I didn’t have that here (4_51)  Q: And what are the things about being here that make you feel normal? What makes you feel happy when you’re here?  A: Yes. The first thing, oh this make me feel good, it’s that over here they care about my kid’s health, they care about the vaccines. They care about taken care of. They care about their education. The first thing they did, they put them in school, they start teaching them English and following up with them. It’s like one – one-to-one, care, which I really admire (4_51)  Q: how do your kids feel about coming here?  A: They are so, so happy. Very, very happy. Because when they were in Jordan, we couldn’t even let them go out to play outside, the neighbor’s kids or something like that, because they all use to say, well, you’re Syria, you’re a refugee, this and that and [inaudible 00:09:32]. Here they can go out, they can play at the park, they can play with their bikes. They got along very very well at school. They don’t even want to stay home on the weekends. They want to go to school, because they love it (4_51)  Q: What are some of the things that have made your transition coming here easier, what are the things that have supported you?  A: The people who are helping me out with, over here in the United States, that’s what makes me get along very easy. And that transition, make it very – [inaudible 00:10:49. (4_51)  Q: is there anything that made your transition to the US easier.  A: Yes, ma’am. Since we got to the airport, we got – [inaudible 00:12:35] a residency, they give us afterwards to get our ID, our social security cards. So you feel that you are stable. You are stabilized in your life. It’s not like you don’t know what’s gonna be happening in the future, in the near future or anything like that. They found a job for my husband. They got my kids in school. So all that makes me have a normal life. The transition did not matter. I have a normal life now, not like when I was in Jordan. I was not for sure what was gonna happen next day (4_51)  Q: What are the things that are supporting you to take care of your health while you’re here in America?  R: Well, [inaudible 00:15:02] in terms of my health and stuff like that and everything, and helping the kids too, helping me, with a lot of things. Have a place to stay, a stable place to stay, healthy place to stay, me and my children. The people who help us out to come over here to United States, the organization, they rent us a place for six months and they pay for electric and water and all the utilities. And we got some help too to get some cash in the beginning before my husband got working. They give us Food Stamps to feed our children to give them the stuff they couldn’t get before. (4_51)  Q: Are you doing anything to help learn more English, like taking a class?  A: Yes, ma’am. I’m going to school once a week. But I’m waiting – we gonna have Internet access at home, so I’m gonna start learning English a little bit more by downloading some apps and stuff. (4_51)  Q: And how do your daughters like being in the United States? Are they going to school?  A: Well at first it was a bit challenging for them, especially since it is a different language. But when they started going to school and they are seeing how the teachers are treating them, things are now easier for them. (5_52)  Q: how has your experience been since you’ve arrived to the United States?  A: Yeah. I am getting better day by day. I am learning new things. I am learning about the laws and rules of this place. And I’m not worried (6_53)  Q: What are some things that have made your experience easier since coming to the United States?  A: So well back in Nepal, there are a lot of problems. I use to work as a worker in a building. I had to carry heavy loads and a small amount of money. Even if they don’t wish to give that money, working for the whole day, they will even not give the money for the labor, what I did. In that situation – so I worked and I did, and I grew up, my family over there, it was really a bad life over there. But when I come to United States, I find everything is perfect. I don’t have to worry about anything else, even if I am sick and if nobody is there to give me warm water. I can open the sink and get warm water from there. So these are examples, what I see here. So it is really amazing country, where I am now, and I’m proud of it [crying] (6_53)  Q: How the insomnia and the weight loss – how does that make you feel?  A Yeah. So actually I have the insomnia because I’m just worried, I’m still looking for the [inaudible 00:02:14] job. And I’m trying to lose weight because right after I came from Egypt I work as chef assistant and actually I spend my time at home cooking. So I’m overweight now (7_54)  Q: can you describe to me the struggles that you’ve had with the job?  A: So actually, the organization was able to find a job for me. But again, to go to the job takes me like two and a half hours. The job [inaudible 00:03:27] and I did just work for two days and then two days after I was very sick. And that’s why I have insomnia because I wanted to have a job (7_54)  Q: Is there anyone in particular whose advice you trust when you have questions about your health  A: So actually, no, there is no specific one. Actually my health is very good. It’s only the insomnia. And back in Egypt I didn’t have this problem, because in Egypt you can just survive a month or two without having money to pay for your rent, you can just borrow that much money, the thousand pounds, from a [inaudible 00:08:31] friend or from an Egyptian friend and it’s just easy. Here, in the states, it’s much, much more difficult. Because no one is helping, and actually work is that not easy and usually when you find a job it’s far and you get paid very little. So that’s what makes me feel very [inaudible 00:09:00] and I have that insomnia (7_54)  Q: Can you tell me a little bit more about your insomnia? Is it something new, and how it affects your life?  A: So actually, I have experienced different types of insomnia. When I was back in Syria I had insomnia because of – first I was detained in the Syrian prisons and I also – like [inaudible 00:11:33] and we [inaudible 00:11:35]. And in Egypt, there was – it was very safe, but again it’s very crowded and just too hard to find a job. But the good thing about it is it was too easy to get help from people. You can just go to the mosque and you find the person who will volunteer to pay for your rent. Here in the states it’s different. If I don’t pay the rent I’m gonna be evicted by the court. Again, here, I was told that organization paid for a one month deposit and one month of rent. And then they told me that for the month of May I’m gonna have to pay the rent out of my pocket. And now I can’t – it’s only five days away from the beginning of May and I have to come up with a thousand dollars. That’s what makes me unable to sleep and that causes the insomnia (7_54)  Q: When you were in turkey, did you have more support from friends around you?  A: Yeah. So actually, yeah it’s different in Egypt, again, because it’s an urban community, it’s very easy to find a job. Although I don’t get paid that much. But there’s no such problem as to paying rent. I know that you can – I use to [inaudible 00:14:29] and I remember I didn’t get any money for the first half month. Then several months after I wasn’t able to make any payment and he was fine with that. So yes, it’s how it is in Egypt. (7_54)  Q: Do you know what health insurance is?  A: Actually I know that United States here is number one, everything, but I had a bad feeling yesterday that [inaudible 00:22:41] coming over to here. For what point I’m here? I don’t have any job. This country is great, but again, I don’t have a job. But then I just said to myself, no, hopefully things will get better and I’ll be okay. And my question to you – are you a psychiatrist? Because actually I don’t think I need a psychiatrist, I just need a job (7_54)  Q: What was your experience like moving to the United States?  A: So actually I like the American people, they’re very decent, they’re very polite and they’re very practical and very serious people. They’re also very compassionate and very kind. Once they know that you a refugee, they all come to help you. Actually I love the life here in the United States (7_54)  Q: What are some challenges that you experienced when you got to the United States, over the past few months?  A: So actually I’ve been here for a month and ten days, that’s like 40 days. And probably the English language was one of the challenges. But actually I have – I can just deal with different people, I can just talk to different people. I have a very social personality. And again, my main challenge is work, as you know. I would say I know Syrian people who came with me on the same airplane. And they went to a different state, and when they call me, they said like they live in paradise. The benefits they’re getting from their state, where they have [inaudible 00:30:42] the rent, they offered them good jobs. And that’s why I’m just gonna wait for a month or two, if things haven’t changed, then I’m gonna probably have to move to a different state (7_54)  Q: And what has made your experience as a refugee more difficult?  A: More difficult for refugee? Maybe the healthcare, because a refugee come here, doesn’t know how to get in the system, the healthcare. And the places. But now I’m familiar with the transportation so I have no problem. I have GPS and transportation. But everybody comes here for the first time, the roads, places, very complicated. So it’s very important to get GPS (8)  Q: How do you get around usually? And have you had any problems getting here from your home?  A: Transportation. Trains, buses. No. Because I have GPS. I just put the address on GPS (8)  Q: what are other challenges or difficulties you faced since coming here?  A: Okay. Maybe the second is shopping, where to go for shopping, because there are some stores very expensive and others very good prices. So it’s one of the challenges, one must know about it (8)  Q: how did you learn about shopping?  A: Yes. He takes me frequently, go shopping with my family of course. And we are – actually my neighbor is Iraqi too, so he talked to me about his experience in the United States. He’s been here for eight years, so he’s very experienced here, and showed me the way, the stores I can go and make shopping. It’s very good experience too (8)  Q: And what do you wish you knew before coming to the United States?  A: Everything. Everything, yes. The healthcare, study, how to get a job, how to get a degree. Many things. How to get apartment or house to live in. Because very – this stuff are unknown for the people outside the United States. I told you the system [inaudible 00:19:14] very different to our country. So I needed to know many things before coming here (8)  Q: Can you describe your health to me?  A: I said, I’m not sure that we ask these questions to you. Because I am here all by myself so I’m always thinking about life, everything is hard. I don’t know if my family is [inaudible 00:02:52], my friends is [inaudible 00:02:57]. But if you would help me to get a place to stay or something that would distract my mind from thinking about my parents, and my family, that maybe would help my life, would improve my life (9_55)  Q: What are some things that make it difficult for you to stay healthy?  A: Thinking about the future. When I think about the future, I don’t know how to leave, I don’t know how to face the world without any help, without the help of my parents, without the help of anyone. So each time I think about the future, I get very, very depressed. I don’t know how my life will end up being (9_55)  Q: So the agency told you you would have insurance for 3 months?  A: Yes. They told me that I will – that after three months they will stop the health and then I’ll have to take care of myself. I’ll have to find a job and take care of myself (9_55)  Q: What do you think would help you most right now?  A: Right now, what would help me is to find a place to stay that I’ll feel comfortable that I will stop worrying (9_55)  Q: And how do you feel now about your experience upon arriving to the US?  A: some of the things that I envisioned, or that I had hoped for is not how I found it (9_55)  Q: Can you tell me about yourself?  A: I like computers. I was born in Togo. I have a passion for anything that’s technology related (10)  Q: Okay. And I think I heard you say that you like to play sports as well. What kind of sports do you like to play?  R: Soccer. I like football and combat sports. (10)  I: Okay. And have you been playing sports since you’ve arrived here?  R: No. First of all, I don’t have the equipment. Second, maybe when I get a job I’ll be able to do something, and maybe I’ll be able to pay for a gym membership (10)  Q: And what was your experience moving to the United States like?  A: I would say surprised. It’s new. And at the same time the reality is different than what we were thinking. I’m trying to adapt (10)  Q: What is different than you expected?  A: Okay. So first of all the university is expensive, and we were looking at it as a paradise. But everything you have to fight for. And also even for the phone, I thought I can just get a SIM card to put in a phone so I can use it. But everything is a bureaucracy (10)  Q: What other challenges have you faced?  A: I would not say that I’ve had a lot of difficulties. I’m not complaining. But I wanted to have maybe the following: I’m waiting for social security. And after getting social security card I would like to get a little job so I can take care of myself, just to be independent (10)  Q: And what has made your experience, since moving here, easier?  A: I’ve benefitted from a lot of things. I have a free Internet connection. I have the possibility to go to the library to get movies and watch them. I would not complain. It’s still better than togo (10)  Q: what did you learn about the US prior to arriving in the US??  A: That you have to be independent. You have to create opportunities for yourself. You have to move. And for university, also now, I’m learning English and maybe I can find a little job for one year or something like that. For university, the tuition is not like Africa. You have to pass a test and also there’s also ways for payment (10)  Q: What are some things that help you stay healthy, that make it easier to take care of yourself?  A: It is the food. I mean, everything I eat here in this country has a ratio, a percentage of sugar or hormones, which is bad to me, personally. Currently I’m living solely on vegetables. Despite the fact that I can buy only small amount of vegetables. They are expensive. I cannot afford buying a lot of vegetables (11_57)  Q: What are some other things that make it difficult for you to take care of your health?  A: Well, bad news. Of course if anybody hears bad news, that is going to affect their health in a bad way.  Q: Can you just tell me a little bit about what your experience was like coming to the United States when you first moved here  A: I when I got here the experience that I got I was able to find that the health insurance – I mean the health system is really good. The living standard is good. The education system is very nice. And also I was a bit scared before because I heard that work – the job, working in the United States is hard because you have to stand all the time for a long time. So I was a bit nervous about it. But when I started working it wasn’t too bad compared to some areas in Africa (13_91)  Q: When you first arrived here, did you – did the organization that helped you did they tell you anything about healthcare or any of the resources or services available to you as a refugee  A: Yes, they help with the food stamps and with the health coverage – health insurance and they help me for the rent for few months. And they just – give us a check lately – last month (14_44)  Q: what are the challenges you faced when you first arrived?  A: Different culture, different language – definitely is a challenge. I wish if I had learned the language before I came here. That would be helpful. (14_69) |
| Values/Priorities/Emotions/Family |  | Q: So you said you didn’t have a normal life or childhood, can you tell me how that affects you  A: Because of all these things that I have been through in my life and then I got married and had these kids, and I thank God for them, I’m not saying anything, but they have more responsible [inaudible 00:02:09] problems added to my life. And it’s all stressing me. (3_48)  Q: Can you tell me a bit more about what’s stressing you out  A: It’s because of my situation, my country situation in Syria and my kids and their problems. These all are problems that I’m thinking about (3_49)  Q: And can you tell me how all of those things, how they make you feel with dealing with all of these?  A: Okay. So of course it is stressing me out. But it’s not that I’m complaining. It’s okay, because everything is a gift from God, even the children are. So I always try to say thank God for everything, even though it is (3_49)  Q: What are some things that you do to help you stay healthy?  A: Well, every time I see my children and I bring them to the school and I pick them up, I feel that I am living some normal life and something that is normal going on in my life. So I will feel a little bit of happy (3_49)  Q: what are the things that make it difficult for you to take care of your health?  A: Well besides the problems of my children and also being far away from my family there in my home country, these are the two problems mainly affecting my health (3_49)  Q:What are some things that you think would make it difficult for you, when you’re sick, to get care?  A: Okay. So I don’t think there is some difficulties in there. But there is some priorities. My children, my daughter and son are my priorities right now. I want them to get better and they are very important for me, even more important than myself. So this is the number one for me and I want to really prevent them to go through the same things that I went through when I was little, like not getting to school or getting educated or this stuff. I will have to raise them for a better way. Yes. I think that I love them more than anything in the world and I want to give them the things that I lost in my life (3_49)  Q: Can you tell me how your children’s health conditions affect you  A: Well, I’m so uncomfortable and unhappy because of my children, but everything else here is perfect. The healthcare, the – every care here is okay for the children and for the adults. But it’s just for me, it’s my children’s condition is really stressing me (3_49)  Q: how did your family feel when they first learned you were coming to the US?  A: They felt the same. Also, they felt the same because of the other daughters, that they didn’t finish their school there and they were suspended for years because of the situation. So they will resume their education, the school here, and it will be better (3_49)  Q: So what are some things you like to do?  A: Most important thing in my life, the things I like to do is take care of my children, see them growing, healthy and just to share with them the moments, spending time, everything like that, go out to dinner with them, with my husband, make them food they like. That’s what I got in my life (4_51)  Q: So how did you feel when you learned you were coming here?  A: Yes, ma’am. I did feel tense a little bit, I did. You coming to a place you don’t know nothing about, you don’t know the culture, you don’t know nothing. So I was a little tense about that (4_51)  Q: And what were your first impressions about the United States, once you got here?  A: The first thing I felt when I got here to United States is that I’m a human again and I’m normal and I’m living again. It’s not like when I was Jordan. Everybody use to just point at you, like a refugee from Syria. I didn’t have that here (4_51)  Q: And what are the things about being here that make you feel normal? What makes you feel happy when you’re here?  A: Yes. The first thing, oh this make me feel good, it’s that over here they care about my kid’s health, they care about the vaccines. They care about taken care of. They care about their education. The first thing they did, they put them in school, they start teaching them English and following up with them. It’s like one – one-to-one, care, which I really admire (4_51)  Q: how do your kids feel about coming here?  A: They are so, so happy. Very, very happy. Because when they were in Jordan, we couldn’t even let them go out to play outside, the neighbor’s kids or something like that, because they all use to say, well, you’re Syria, you’re a refugee, this and that and [inaudible 00:09:32]. Here they can go out, they can play at the park, they can play with their bikes. They got along very very well at school. They don’t even want to stay home on the weekends. They want to go to school, because they love it (4_51)  Q: is there anything that made your transition to the US easier.  A: Yes, ma’am. Since we got to the airport, we got – [inaudible 00:12:35] a residency, they give us afterwards to get our ID, our social security cards. So you feel that you are stable. You are stabilized in your life. It’s not like you don’t know what’s gonna be happening in the future, in the near future or anything like that. They found a job for my husband. They got my kids in school. So all that makes me have a normal life. The transition did not matter. I have a normal life now, not like when I was in Jordan. I was not for sure what was gonna happen next day (4_51)  Q: So what are the things that make it difficult to take care of your health?  A: There’s nothing keeping me from keeping myself healthy. I would like just to get – to go to a place to do some gym and stuff like that. But just me, [inaudible 00:17:21] my children, a lot, I want them to get use to everything, I want to be there for them. I don’t take care of this – but it’s not a big deal, as long as we’re happy. But my health is fine. I don’t have no problems (4_51)  Q: What might happen if you get sick  A: If it’s something simple, we take care of each other, my husband, me and the kids, we take care of each other. If it’s something easy and something we can take care of. But just like my husband, he got some problems with something [inaudible 00:22:43] lately, had to call 911 and we need to go to the hospital. And you know, the stuff we cannot take care of, we go tot eh doctors. That’s it. And we take care of each other. That’s it (4_51)  Q: Tell me about yourself  A: Well, my name is [inaudible 00:01:25] and I’m a mother for three. And since I came to the United States, we started taking our son, who has been diagnosed with blood cancer, to the hospital. And he’s been in the hospital since then. I switch places with his father. Sometimes the father stays with him and sometimes I stay with him, because we still have two daughters as well waiting for us at home. So when they need me, I go there, and the father will come stay with our son, and vice versa. So this is our life now. I just thank God for everything happening, for good and bad. I just pray that my son will get healed soon. And all together, as a family, we will be in one (5_52).  Q: what are some things that help you take care of your own health and the health of your children?  A: Actually right now, all I’m thinking about is my son and for him to get well. I don’t know what to tell you about the rest. But even my daughter – one of my daughters, she has very weak vision, only – on the scale she only has one degree, so she can’t really see well at all. She needs someone to take care of her all the time. (5_52)  Q: how did you feel when you learned that you were coming to the United States?  A: Yes. Actually I was really happy. And I think of that – when I heard we was coming to United States, that this is a blessing from God. This is proof that he is happy with us. And maybe he will continue and heal my child (5_52)  Q: how did you feel when you learned that you were coming to the United States?  A: Yes. Actually I was really happy. And I think of that – when I heard we was coming to United States, that this is a blessing from God. This is proof that he is happy with us. And maybe he will continue and heal my child (5_52)  Q: What made you excited to think about coming here?  A: Well I was really optimistic about this, when someone knows he’s going to the United States with all the healthcare that’s being provided over there, it’s different. So that’s why we were so happy (5_52)  What have been the barriers to taking care of your children or yourself?  R: Actually none. The only thing that we’re facing right now is my son’s sickness. And only I’m thinking about and praying that may God interfere and heal him one day, and my daughter as well, her vision (5_52)  Q: what are some things that make it difficult for you to stay healthy or to take care of yourself?  A: If I have to tell you from the beginning, we got a big family and we supposed to be out of the house. So I went out of the house with my two kids and started leaving separately. And since I don’t have a male – I have two kids and there was nobody to help me. I am the only one who take care of the kids. And I was quite worried about my kids. And I was only one who take care of them and who feed them everyday. So I have to go out to work, for them. And at that time I don’t have time to treat my sickness. So because of that it has been so long that I didn’t get proper treatment (6_53)  Q: can you tell me how that makes you feel [not getting proper treatment in the past]?  A: I feel that when I was alone I don’t feel any sleepy at all at night, so I feel sleepy during the daytime. But in Nepal they think that it is bad to sleep during the day. I feel to sleep outside, but it is not good in our culture that people will – they will not feel good if I sleep outside. So I went to the doctor and they gave me sleep pills. And that pill helped me to sleep and I sleep in a good way to seven months. And after seven months again, my kids they grew up and when they were not at home, I started feeling the same thing, no sleep at night. When the kids are at home, then I feel comfortable and I sleep. When the kids are outside, I don’t feel any sleepy. So in this way I spend my days. (6_53)  Q: Are there any things that help you take care of your health, make it easy for you to take care of your health?  A: About my health, there is nothing to do now, if anybody speaks louder I feel so scared. And if I heard any loud noise that makes my heartbeat so strong and I feel so scared. So if my son – if they speak louder at home, that also makes me so scared. I don’t know what is going on. I can give you the example, if you place plastic bag close to the fire, then how suddenly it will become one ball. In that way, my mind will become one ball because it’s scared (6_53)  Q: How do you think that affects your ability to take care of yourself everyday?  A: If there is nothing in my mind, I don’t feel any tension at all, if my kids are well if they are good, then I will do my daily activities. But I feel so weak and tired quickly (6_53)  Q: do you know if you have specific healthcare services or resources that you have access to  A: Yes. I am learning slowly. I am getting some idea from my friends. They told me that I should not have to worry about anything else, I should not have to be scared about anything else. Even [inaudible 00:19:40] should not have to worry about my kids. They will work by themselves. And everything will be fine, I can walk freely outside. So everything looks like very good (6_53)  Q: What was your first impressions of the United States when you arrived?  A: Well, in Nepal, when somebody dies, we need to carry them to the river and [inaudible 00:24:10] and we have to burn the dead body. Even at that time, the [inaudible 00:24:20] were responsible to carry to the riverbank, but they were not doing that. And here, they don’t have to do that if they don’t want. So even when one is alive, he can leave himself freely and even when one has died, you don’t have to worry about who will take me to the riverbank. So these things make me quite happy (6_53)  Q: What are some things that have made your experience easier since coming to the United States?  A: So well back in Nepal, there are a lot of problems. I use to work as a worker in a building. I had to carry heavy loads and a small amount of money. Even if they don’t wish to give that money, working for the whole day, they will even not give the money for the labor, what I did. In that situation – so I worked and I did, and I grew up, my family over there, it was really a bad life over there. But when I come to United States, I find everything is perfect. I don’t have to worry about anything else, even if I am sick and if nobody is there to give me warm water. I can open the sink and get warm water from there. So these are examples, what I see here. So it is really amazing country, where I am now, and I’m proud of it [crying] (6_53)  Q: : Does your wife help you take care of your health at all?  A: Actually, she helps me, I mean she cleans the house and sometimes when she cooks I help her – if I cook, she comes and helps me. And we never – we rarely go out, like before I didn’t have any mood to go out. Sometimes I go to the library, with the Internet and I look for jobs. That’s what I do. (7_54)  Q: In general, whose advice do you trust for taking care of your health?  A: My wife. (8)  Q: And other than smoking are there other things that she talks to you about, about your health?  A: Just smoking. She talks, yes, to me about smoking. Because everything I do is good, unless smoking. (8)  Q: So what were your first impressions when you moved here, what did it feel like to come here?  A: I was very excited when I came here. Because a long time ago I wanted to come here. But it takes a long time for insurance, VISA, my studies too, I needed to finish my studies [inaudible 00:14:30] come here. But I was very excited. I love that place, I love the procedures (8)  Q: And when you first got here, how did you feel?  A: Happy. The first feeling was very tired, because my traveling, four airplanes to reach here from Iraq to Jordan from Jordan to Frankford, Germany and from Germany to Washington and Washington to Philadelphia. So I was very tired. But the next day I was very happy and very excited (8)  Q: Can you describe your health to me?  A: I said, I’m not sure that we ask these questions to you. Because I am here all by myself so I’m always thinking about life, everything is hard. I don’t know if my family is [inaudible 00:02:52], my friends is [inaudible 00:02:57]. But if you would help me to get a place to stay or something that would distract my mind from thinking about my parents, and my family, that maybe would help my life, would improve my life (9_55)  Q: Can you tell me more about things that would help you stay health?  A: By that I mean having a life just to myself, with no parents, no family, I don’t settle, I don’t settle down, I keep thinking about it all the time. That bothers me so much (9_55)  Q: What were some things that made your experience easier?  A: It is quite a number of things. I thought in me, that if I come to United States, I won’t go hungry, because most times I didn’t get food to eat. I had hopes of going back to school. I thought that I would go back to school and be able maybe to have a good life. I also thought I’d be safe, because I didn’t have security. So it’s quite a number of reasons that made me quite hopeful about coming to United States (9_55)  Q: What are some things that help you stay healthy, that make it easier to take care of yourself?  A: It is the food. I mean, everything I eat here in this country has a ratio, a percentage of sugar or hormones, which is bad to me, personally. Currently I’m living solely on vegetables. Despite the fact that I can buy only small amount of vegetables. They are expensive. I cannot afford buying a lot of vegetables (11_57)  Q: Before moving to the United States, did you talk to anyone about healthcare in this country?  A: No  Q: Did you talk to anyone about coming to America?  A: No. Nobody spoke to us about this. But it was the commission if you will, the immigration commission who contacted us and they brought us here to the states (11_57)  Q: Since moving here, what are some things that are surprising to you or things that you weren’t expecting?  A: [inaudible 00:14:30] I’m not sure if this answers your question or not, but he said, well, the living here is different, it’s a different language, different laws, different everything. However, before we came to the states, this organization commission in Egypt, they warned us against it. They said, you’re going to see a new country, new law, new languages, and it is going to be 180 degrees different for you (11_57)  Q: Can you tell me how you first felt when you came to the United States, what your experience was like?  A: It was a surprise. We were surprised by the culture here, how the people are different, how they are organized, how elevated the culture is here, how the streets are clean. It was 180 degrees different than our [inaudible 00:19:00} country (11_57)  Q: How does your wife help you?  A: She’s helping me with the cooking. I mean, the healthy foods and stuff like that (14_17) |
| 1Health Status & Perception | Pre US | Q: Tell me just a little bit more about what you’re experiencing [with regards to your health].  A: Well, I was back in Nepal when my feet were swelling, they said it was because of the sun. And when it is swelling, they sometimes told me that it is because of cold. But they don’t like to treat me over there, UNFCR also doesn’t like to treat me. Now I came here. Now I hope that my sickness will come out and everything will be checked and find out the diagnosis, what is wrong in me and they will find out individually what is going on inside (6_53)  Q: what are some things that make it difficult for you to stay healthy or to take care of yourself?  A: If I have to tell you from the beginning, we got a big family and we supposed to be out of the house. So I went out of the house with my two kids and started leaving separately. And since I don’t have a male – I have two kids and there was nobody to help me. I am the only one who take care of the kids. And I was quite worried about my kids. And I was only one who take care of them and who feed them everyday. So I have to go out to work, for them. And at that time I don’t have time to treat my sickness. So because of that it has been so long that I didn’t get proper treatment (6_53)  Q: can you tell me how that makes you feel [not getting proper treatment in the past]?  A: I feel that when I was alone I don’t feel any sleepy at all at night, so I feel sleepy during the daytime. But in Nepal they think that it is bad to sleep during the day. I feel to sleep outside, but it is not good in our culture that people will – they will not feel good if I sleep outside. So I went to the doctor and they gave me sleep pills. And that pill helped me to sleep and I sleep in a good way to seven months. And after seven months again, my kids they grew up and when they were not at home, I started feeling the same thing, no sleep at night. When the kids are at home, then I feel comfortable and I sleep. When the kids are outside, I don’t feel any sleepy. So in this way I spend my days. (6_53 |
| Health Status & Perception | Post US | Q: How would you describe your health? (1_47 line 44)  A: I’m in good health (1_47 line 49)  Q: What makes your health good? 1_47 line 52-53)  I do know that my general health is in good condition. But on my first visit here, I had to undergo a screen – health screening, and then the doctors did the – and on the second visit, they let me undergo some tests, and the results showed that I was okay. So that makes me feel – believe that I am in good health. And also, I also feel good in my general well-being, so I’m pretty sure that I’m in good health (1_47 line 54-58)  Q: What helps you stay healthy? (1_47 line 59)  A: I was following the guidelines. For example, they asked me to quit smoking, so I watch my smoking. So I’m pretty sure that I am maintaining a good health (1_47 line 60-61)  Q: what makes it difficult to take care of your health? (1_47 line 62)  A: There are no hurdles to my route to the good health (1_47 line 63)  Q: What are things that help you take care of your health (1_47 line 64-65)  A: I don’t drink at all, and then I would choose my cigarette smoking. Like if I smoked five cigarettes a day, now I will reduce it to two or three. And then also, I no longer do the practice of beef for chewing. So that will – that should help maybe my good health (1_47 line 66-68)  Q: . How would you describe your health  A: Okay. Generally speaking I’m a healthy person. I’m very energetic (2_48)  Q: What are certain things that help you stay healthy  A: Okay. I do consider diet and my nutrition very important, I take into my consideration. I go for a walk or I walk for almost two hours per day. Also I drink lots of water (2_48)  Q: Are there any things that get in the way of you doing those things and staying healthy  R: No. I don’t think so (2_48)  Q: how would you describe your health to me  A: It’s about medium health (3_49)  Q: What are some things that you do to help you stay healthy?  A: Well, every time I see my children and I bring them to the school and I pick them up, I feel that I am living some normal life and something that is normal going on in my life. So I will feel a little bit of happy (3_49)  Q: what are the things that make it difficult for you to take care of your health?  A: Well besides the problems of my children and also being far away from my family there in my home country, these are the two problems mainly affecting my health (3_49)  Q: What are the things that are supporting you to take care of your health while you’re here in America?  A: Well, [inaudible 00:15:02] in terms of my health and stuff like that and everything, and helping the kids too, helping me, with a lot of things. Have a place to stay, a stable place to stay, healthy place to stay, me and my children. The people who help us out to come over here to United States, the organization, they rent us a place for six months and they pay for electric and water and all the utilities. And we got some help too to get some cash in the beginning before my husband got working. They give us Food Stamps to feed our children to give them the stuff they couldn’t get before. (4_51)  Q: So what are the things that make it difficult to take care of your health?  A: There’s nothing keeping me from keeping myself healthy. I would like just to get – to go to a place to do some gym and stuff like that. But just me, [inaudible 00:17:21] my children, a lot, I want them to get use to everything, I want to be there for them. I don’t take care of this – but it’s not a big deal, as long as we’re happy. But my health is fine. I don’t have no problems (4_51)  **Q: Who do you talk to about your health and who do you get your information from about how to stay healthy?**  **A: There is one person that if we need anything, anything that has to do with our health or anything, we go to him. He’s in an organization. He gives us the schedule to go to the doctor if we need to or something. But I guess that’s the person I can go to if I need anything, appointments with doctors or something or any advice. Other than that, when we came over here to United States, they check me out. I went to the doctors and everything and everything was fine. I think if something was wrong they would have just told me by now (4_51).**  Q: what are some things that help you take care of your own health and the health of your children?  A: Actually right now, all I’m thinking about is my son and for him to get well. I don’t know what to tell you about the rest. But even my daughter – one of my daughters, she has very weak vision, only – on the scale she only has one degree, so she can’t really see well at all. She needs someone to take care of her all the time. (5_52)  **Q: Are there any things that help you take care of your health, make it easy for you to take care of your health?**  **A: About my health, there is nothing to do now, if anybody speaks louder I feel so scared. And if I heard any loud noise that makes my heartbeat so strong and I feel so scared. So if my son – if they speak louder at home, that also makes me so scared. I don’t know what is going on. I can give you the example, if you place plastic bag close to the fire, then how suddenly it will become one ball. In that way, my mind will become one ball because it’s scared (6_53)**  Q: How do you think that affects your ability to take care of yourself everyday?  A: If there is nothing in my mind, I don’t feel any tension at all, if my kids are well if they are good, then I will do my daily activities. But I feel so weak and tired quickly (6_53)  Q: Can you describe your health to me?  A: Actually yeah, good. It’s good, somewhat good. And actually I have some insomnia and I’m trying to lose my weight (7_54)  Q: How the insomnia and the weight loss – how does that make you feel?  A Yeah. So actually I have the insomnia because I’m just worried, I’m still looking for the [inaudible 00:02:14] job. And I’m trying to lose weight because right after I came from Egypt I work as chef assistant and actually I spend my time at home cooking. So I’m overweight now (7_54)  Q: Is there anyone in your life that helps you take care of your health?  A: Actually my wife. She does clean the house, and I cook (7_54)  Q: : Does your wife help you take care of your health at all?  A: Actually, she helps me, I mean she cleans the house and sometimes when she cooks I help her – if I cook, she comes and helps me. And we never – we rarely go out, like before I didn’t have any mood to go out. Sometimes I go to the library, with the Internet and I look for jobs. That’s what I do. (7_54)  Q: Is there anyone in particular whose advice you trust when you have questions about your health  A: So actually, no, there is no specific one. Actually my health is very good. It’s only the insomnia. And back in Egypt I didn’t have this problem, because in Egypt you can just survive a month or two without having money to pay for your rent, you can just borrow that much money, the thousand pounds, from a [inaudible 00:08:31] friend or from an Egyptian friend and it’s just easy. Here, in the states, it’s much, much more difficult. Because no one is helping, and actually work is that not easy and usually when you find a job it’s far and you get paid very little. So that’s what makes me feel very [inaudible 00:09:00] and I have that insomnia (7_54)  Q: Can you tell me a little bit more about your insomnia? Is it something new, and how it affects your life?  A: So actually, I have experienced different types of insomnia. When I was back in Syria I had insomnia because of – first I was detained in the Syrian prisons and I also – like [inaudible 00:11:33] and we [inaudible 00:11:35]. And in Egypt, there was – it was very safe, but again it’s very crowded and just too hard to find a job. But the good thing about it is it was too easy to get help from people. You can just go to the mosque and you find the person who will volunteer to pay for your rent. Here in the states it’s different. If I don’t pay the rent I’m gonna be evicted by the court. Again, here, I was told that organization paid for a one month deposit and one month of rent. And then they told me that for the month of May I’m gonna have to pay the rent out of my pocket. And now I can’t – it’s only five days away from the beginning of May and I have to come up with a thousand dollars. That’s what makes me unable to sleep and that causes the insomnia (7_54)  Q: So what are some things that help you stay healthy?  A: Being in a place where I am not worried and thinking about everything, that would be good for me (9_55)  Q: What are some things that make it difficult for you to stay healthy?  A: Thinking about the future. When I think about the future, I don’t know how to leave, I don’t know how to face the world without any help, without the help of my parents, without the help of anyone. So each time I think about the future, I get very, very depressed. I don’t know how my life will end up being (9_55)  Q: Are there any things that make it easier or help you to stay healthy?  A: Yes. If I got a place, a nice place to stay, or a place where I’d have help, I’d have – so that I’m not thinking about life just by myself, being alone without having anyone to call on or anyone to talk to, that would be good (9_55)  Q: Why don’t you feel comfortable in the place that you’re staying right now?  A: I worry so much because when I fall sick I have no one to take care of me. I do not have anyone. I think about my parents so much. And then I think about so much, that after two months they’re still paying for me, and then I worry that I won’t have a place to go because now I am falling sick (9_55)  Q: and what are some of the things that help you take care of your health?  A: Right now, I need to do sports, because they told me about my cholesterol being elevated. So I will do my best to find shoes, sports shoes maybe next week so I can start doing sports (10)  Q: And have you been sick here at all yet  A: no (10)  Q: Do you have any questions for me, or anything else that you’d like to talk about?  A: If I have a question concerning tuberculosis, is it serious, can it affect a lot of part of my body? (10)  Q: can you tell me about your health?  A; So my health is poor. I have diabetes. I have [inaudible 00:00:35]. I have [inaudible 00:00:37] I don’t know what he means. And I also have a problem in my nerves. I have a problem in my disc. My whole body hurts. And now I’m sitting on the chair, but I can barely tolerate sitting on the chair (11_57)  Q: What are some things that help you stay healthy, that make it easier to take care of yourself?  A: It is the food. I mean, everything I eat here in this country has a ratio, a percentage of sugar or hormones, which is bad to me, personally. Currently I’m living solely on vegetables. Despite the fact that I can buy only small amount of vegetables. They are expensive. I cannot afford buying a lot of vegetables (11_57)  Q: When you have questions about your health, where do you go?  A: You’re off topic sir. I’m sorry Ma’am, I had to – he just went two ways because he was [inaudible 00:05:43]. He said, well, ten days ago I felt that my sugar level was down, so I went to the dentist. It was the people at the dentist office who measured my sugar level and then they referred me to the hospital. I don’t have a machine to measure my sugar level. I’m not working. Yes, I have medications, but I’m not sure if I’m doing well with them or not. And for the nutrition, like I said earlier, the food here has hormones and [inaudible 00:06:07] and stuff, and it’s bad for me (11_57)  Q: Can you tell me about how that makes you feel? How does that affect you, all of these problems?  A: Well, because of the thing I have, and the tiredness I feel and not being able to lift my feet or legs, I spent half of the day sleeping (11_57)  Q: And do you know how long your health insurance will last?  A: They gave me that information. But because of my diabetes, I have been forgetful (11_57)  **Q: What were some things that made your experience coming to the US easier?**  **A: Okay. So speaking about going to the doctor or going to the hospital, it is much better than before. It even makes you feel psychologically better and comfortable, because they treating you better here (11_57)**  Q: Can you describe your health to me?  A: Well my health was the [inaudible 00:01:22] when I was in Africa they tested me and then they found me with syphilis. And which I think that’s what they’re trying to see right now. And also in addition to that I think it’s because of the weather change because I get so – I sweat a lot at night. I can wake up almost four or five times a night to take shower. So it’s – I don’t know if it’s because of the hot season – I don’t know. But yeah that’s all I have I would say (13_5)  Q: ? Is there anything you do every day that you do to take care of yourself – to take care of your health  A: When I – Africa I used to play ball often – soccer. **And when I got here I tried to play soccer three times and I stopped. I was kinda discouraged a little bit because I didn’t have the necessities as far as the shoes to go play with and all those things**. So I was kinda bit discouraged. I was just kinda bit discouraged by all that – the fact that I didn’t have. I went and played a couple – like four times and – but I wasn’t able to afford the shoes and all that and decided to stop (13_18)  Q: Is there anyone in your life who helps you take care of your health?  A: Yeah, my wife, sister and mom (13_32)  Q: If you have any questions aout your health, who do you ask?  A: Well I might talk to my wife or mom or dad. They all stay here (13_36)  Q: Can you describe your health to me?  A: I don’t have any health issues except for poor vision (14_3)  Q: Do you think you’re healthy?  A: They told me I have diabetes, but I am not sure. I don’t know (14_5)  Q: what are some thing that help you stay healthy?  A: I just try to eat a healthy diet because of the diabetes and I take the medication they told me to take. And that’s it (14_7)  Q: Are there any things that make it difficult to take care of your health? A: No (14_11)  Q: Is there anyone in your life that helps you take care of your health?  A: Me and My wife (14_13)  Q: Can you describe your health to me?  A: It’s good. I am healthy (15_2)  Q: what are some thing that help you take care of your health?  A: I eat on time. I take showers. Clean up. That’s how (15_6)  Q: is there anyone who helps you take care of your health?  A: my husband. He helps me clean up, he helps me with everything (15_12)  Q: Can you tell me about your health and what helps you stay healthy  A: My health is good. I exercise and things (16_2)  Q: is there anyone in your life who helps you take care of your health?  A: Family, my cousings. They just help me out to get good food – get good, healthy food and good [inaudible 00:01:55] and everything. (15_8) |
| Knowledge, Understanding & Perception of US healthcare system |  | Q: what would you do if you were more sick, and you couldn’t take care of yourself? (1_47 line 134-135)  A: Yes, if this is – this sounds like not a – I mean, if this sounds like an – a real illness, I would go to a clinic (1_47 line 136-137)  Q: Sometimes people get so sick they need to see a doctor right away, but clinics – doctors at the clinic can’t always make an appointment the same day. So what would you do in that situation (1_47 line 138-140)  A: I’ve never encountered anything like a major illness so far. So I have no idea what I would do in such a situation, but I have no – I don’t know how to answer this question (1_47 line 141-142  Q: And what health services or resources should you get in the United States because you’re a refugee (1_47 line 149-150)  A: everybody, whether you – whether or not you are a refugee has the same opportunities or same rights to get medical (1_47 line 151-153)  Q: Do you know the hospitals that are near you, or would you know where to go, which hospital to go to?  A: Okay. I hadn’t thought actually to search for the nearest hospital. But only know if I was in that situation I would definitely find the nearest hospital (2_48)  Q: Do you know any resources right now that you have access to in the United States  A: No (2_48)  **Q: do you know what health insurance is**  **A: Is it life insurance (2_48)**  Q: And now, if you were to get sick here, in the United States, where would you go?  A: Of course, the doctor (3_49)  **Q: Do you know what health insurance is?**  **A: They told me that it’s something for free. They told me that it’s providing medication and treatment and that the state is provided it to the people (3_49)**  Q: Who is it that told you about health insurance?  A: The resettlement organization (3_49)  Q: Did the organization tell you for how long you have insurance or how long you need it for?  Q: they told me but I forgot (3_49)  so if you ever have any questions about your health or about healthcare, who do you ask or where do you go to?  A: I don’t know (3_49)  Q: Are there any specific services or resources in the United States that you wish you had that are healthcare related, that you wish you had access to?  R: No. I don’t know (3_49)  Q: Is there anything that you wish you would have known about healthcare in the United States before coming to the United States?  A: No. I didn’t know. That they will take care of my children (3_49)  Q: how did you feel when you first heard that you were coming to the United States?  A: I was so happy, because it’s the first country in the medical point of view. I was really happy and thrilled (3_49)  **Q: And do your husband and children, do they have a regular doctor who they see all the time?**  **A: Yes. My husband called the – yes, we do have a family doctor. And my husband called the number we got on the card for my children too. But at least they should have just given us the same day for a sick child, or next day, to be seen. But they didn’t. (4_51)**  Q: So what are some things, that, when you’re talking to doctors or trying to schedule appointments, what are some things that make that easier for you?  A: When we first came here to United States and they discovered what’s going on with our son, they took him in immediately. And he didn’t leave the hospital since then. Tell you the truth, the doctors here are really nice. They give you proper time, and explain things and the way they deal with the person is really very nice way, I feel really comfortable with them. As for my doctor, her father, he is the one who is taking care of all the appointments and scheduling them. So mainly he’s the one who is dealing with this thing (5_52)  Q: So do you feel that you are a healthy person?  A: By looking from outside, I am fine. But inside, I have a lot of pain. My vision is getting lost and I can’t hear properly and I have pain in all of my parts in the body. And I have pain on my joints. And I feel like my abdomen is getting bigger and bigger. I was thinking to have an ultrasound, but I haven’t done that yet, because of all this I am feeling (6_53)  Q: Tell me just a little bit more about what you’re experiencing [with regards to your health].  A: Well, I was back in Nepal when my feet were swelling, they said it was because of the sun. And when it is swelling, they sometimes told me that it is because of cold. But they don’t like to treat me over there, UNFCR also doesn’t like to treat me. Now I came here. Now I hope that my sickness will come out and everything will be checked and find out the diagnosis, what is wrong in me and they will find out individually what is going on inside (6_53)  Q: So now, if you got sick, where would you go, living in the United States?  A: Here in the United States, I come to hospital. The people, they will tell me the appointment. I have appointment here, so I come here, they will tell me that (6_53)  Q: Is there anyone in particular you ask or any resource that helps you answer any questions?  R: I have no idea. I am here only for 31 days now (6_53)  **Q: Do you know what health insurance is?**  **A: I don’t know. I think it is a paper from the hospital (6_53)**  Q: do you know if you have specific healthcare services or resources that you have access to?  Q: As a refugee and new citizen to the United States, were you told about any health resources that are available to you?  A: Yes. I know the organization has offered me eight months of coverage and also that may be extended to a few more months. But then eventually it will expire. Again, it’s a health coverage, but again, there’s no job (7_54)  Q: what makes it difficult for you to take care of your health.  A: There is nothing. I use to smoke. The only problem I have. I’m trying to quit now. It’s difficult. But I think I can, in the future I will have quit. Gradually. (8)  Q: What sorts of things do you think will help you to quit smoking?  A: I think a prescription may help. Also practicing, like workout (8)  Q: Does anyone like your friends and family help you to quit smoking or help you with anything else in your –  A: Yeah. They keep advising me all the time. And nobody here smoke, family or friends. So it’s only me who smokes. Sometimes I feel lonely because I’m the only one smoking. I think this helps too (8)  Q: What are the things that help you take care of your health? So what keeps you healthy?  A: Healthy foods. Work outdoors sometimes, yes. Outdoor sports. (8)  Q: So right now you would like to know more about what coverage you have under your current health insurance  A: yes (8)  Q: Now in the US, what would you do if you became sick?  A: Actually, yes, different, very different. I don’t – I mean, I think the only place to go is the hospital when I get sick or one of my family get sick. Because I don’t know doctors. I don’t know private clinics (8)  Q: so as a refugee and a new citizen here, what health resources are available to you?  A: I don’t know what is available to us (10)  Q: did you receive information at all about healthcare services when you got here?  A: So, yes, when we arrived, somebody talked to us about that. They told us that there is an insurance available to us for eight months and after eight months we will have to manage ourselves to pay for our health insurance (10)  Q: so what information do you wish you had about healthcare in the United States?  A: I think I have the necessary information (10)  Q: What do you understand about health insurance?  A: So health insurance covers the fees for if we get sick. And it gives us access to a doctor. So if there is an emergency, we have to go to the emergency. If we’re sick, we go to the doctor. And for preventative appointments, we need to make appointments for them (10)  Q: what are some things about healthcare in the US that are surprising or that you didn’t expect?  A: I would say it’s different. Maybe organized, but it’s not surprising (10)  Q: at some point, HIAS will stop helping you and you will have to do these things for yourself. Do you know how you will manage to arrange that?  A: Yes. HIAS will stop helping us when we know more. So they will show us before letting us do things ourselves (10)  Q: what are some of the things regarding helath that you think you need more help with from them (HIAS)  A: For example, if I’m sick, who should I contact, which hospital should I contact? And they already chose health insurance for us. What are the advantages of the insurance that they chose for us, and how we can benefit from it fully (10)  Q: do you know what health insurance is?  A: Something to insure the costs and visits for hospitals and pharmacies and so on (11_57)  Q: Do you know if you have health insurance?  A: Yes. I do have one (11_57)  Q: For how long have you had insurance?  A: I would say I’ve had it for a few weeks (11_57)  Q: How is money important for taking care of your health?  A: Of course money is important. If we go to the clinic you have to pay money. Medicine at the pharmacy you have to pay money. To be able to make money you need to be healthy. So health is more important, the most important (12_6)  **Q: In the United States, the healthcare system is complicated and can be confusing for many people. I would like to learn a little bit about what you’ve learned so far**  **A: If you get sick, like normal, like cold or coughing or running nose, you go to the pharmacy and buy the medicine and take care of yourself. You cannot go to the big hospital when you just get sick normal [inaudible 00:07:22]. But when you get sick [inaudible 00:07:27] really sick you need to go or make an appointment. And then when you got an appointment you go see the doctor at the big hospital. (12_31)**  Q: And as a refugee, what resources are available to you?  A: disease you come with into this country, they will give you the treatment. You will be cured. (12_39)  Q: Did you receive information about healthcare when you arrived?  A: yes, They tell me if you don’t have the benefits [inaudible 00:09:18] benefit, then it’s very difficult to go see a doctor at the clinic or hospital. (12_44)  Q: And can you tell me more about your healthcare benefit and your health insurance? What do you know about it  A: **However you think [inaudible 00:10:16] you cannot go buy the medicine at the pharmacy by yourself. You need permission from your doctor. And when the doctor approves you, you can buy the medicine at the pharmacy.** (12_48)  Q: did you talk to anyone when you were in Malaysia about the US healthcare system?  A: **Yes, They tell me that if you have – your benefit expire, you be in trouble. It’s very difficult to get treatment (12_53)**  Q: And what are the things about healthcare in the US that were surprising to you or that you weren’t expecting  A: Only one thing. **Without an appointment you cannot see a doctor. Whatever you are, how serious you are and you are sick, and that made me really, really surprised in why I cannot see a doctor, why I’m sick and I need to see a doctor but I cannot get to see a doctor without an appointment. That made me really surprised** (12_57)  Q: Do you know what to do in order to prevent your health insurance from running out?  A: I didn’t learn about that yet (12_61)  Q: what do you wish you knew about the healthcare system in the United States  A: I have so many questions. What I mean is whatever I need health information or whatever I want, there is a law in US, they’re gonna abide by the law to follow by the law. So I have to follow by the law too. Whatever I want I have to look at the rules and regulations in the US. (12_73)  Q: Now in the US, where would you go if you became sick  A: The hospital (13_45)  Q: do you know where the hospitals are close to your house?  A: no  Q: How would you figure out how to get there?  A: Yeah well I request for an address. I mean it depending on what I’m – what the – this problem I have. So I would always get an address or I would request for an address where that hospital is (13_51)  Q: When you first came here did anyone – including the agency that helped you – talk to you about any healthcare services or resources that you have access to  A: Well I was told that if I have any problem with the illness I can go to the doctor and if it’s far I can always go to the pharmist and they should be able to assist me and I can buy medicine from there (13_56)  Q: Did they tell you anything else about what to do if you became sick or where to go, who can help  **A: Well I was told that if I happen to have any kind of strong type of illness or if I get some type of accident, if I cut myself and I can – I’m not able to walk, I’m not able to do anything I can call and be arrange to come and help me. And if it’s something that I can handle myself I can – I could always go to the pharmist and buy some medicine (13_61)**  Q: Can you just tell me a little bit about what your experience was like coming to the United States when you first moved here  A: I when I got here the experience that I got I was able to find that the health insurance – I mean the health system is really good. The living standard is good. The education system is very nice. And also I was a bit scared before because I heard that work – the job, working in the United States is hard because you have to stand all the time for a long time. So I was a bit nervous about it. But when I started working it wasn’t too bad compared to some areas in Africa (13_91)  Q: Now in the US, where whould you go if you became sick?  A: I have to go to the hospital (14_33)  Q: Do you know any hospitals close to your house?  A: : I don’t know any hospital close to me, ma’am (14_35)  Q: how would you get there?  A: I don’t know what to do, ma’am – I’m not gonna lie to you. I just got here two months ago (14_37)  Q: Do you know what health insurance is?  A: I’m not quite sure about the name. Yeah, he told me that if I get sick or something they can cover my – me seeing the doctor or [inaudible 00:05:37] like that (14_53)  Q: Do you know how long it lasts for?  A: Yeah, they told us that as long as I don’t work, I can benefit from this program. But if I start working, I can’t benefit from this program (14_56)  Q: If you ever have questions about your health, do you go online or read books?  A: I read sometimes. I don’t have internet, so I cannot go to that (15_21)  Q: now in the US, where would you go if you became sick?  A: The hospital (15_33)  Q: And when you first arrived here, did anyone talk to you about the healthcare resources available to you  A: Yes. I heard. They told me (my sister) that if I’m sick, then they’re going to check it. The doctor is going to check it. They told me that like in Nepal, they check when we are sick. They, over here too, we can go to the doctor, and they check it. (15_55-60)  Q: do you know what health insurance is  A: I just know that if you have insurance, then doctor check and you can get the medicine free (15_63)  Q: Do you know if you have it?  A: yes, I have it for 8 months. After eight months, you have to make another one (15_65)  Q: Who told you that you have insurance?  A: back in Nepal in the orientation class (15_78)  Q: Did anyone tell you what healthcare would be like in the US?  **A; In Nepal, you don’t have to make an appointment to go to doctor, but over here, you have to make a doctor appointment when you go to doctor. (15_86)**  Q: So, if you have a medical question, what do you do? Do you go to the doctor? Do you look online? Do you ask somebody  A: No. I just come to the hospital, ma’am, and see a doctor – see somebody professional (16_18)  Q: If you were really, really sick and you didn’t think you could wait for an appointment, where would you go  **A: I have to call 911 (16_32)**  Q: Do you have health insurance:  A; yes. They tell me that I have health insurance for eight months and anything – I can go to the doctor and see the doctor for anything – if I get sick or something (16_52)  Q: what happens after 8 months?  A: I have no idea what I'm gonna do after that. I have to have a job and maybe get insurance from them. (16_57) |
| Knowledge, Understanding & Perception of US healthcare system | Barriers | Q: Are there any people that help you with your health?  A: No. I don’t have nobody. Yeah, I don’t have any friends, I don’t have any family, family members over here. So just the end of this month gonna be six months since we’ve been here. So it’s so new. (4_51)  Q: And what information do you wish you had about healthcare in the United States?  A: Actually, just in general, I’d just like to know more about this – knowledge would be a good thing, knowing more about the healthcare system (5_52)  Q: What specific parts of the healthcare system would you like to know more about?  A: Actually in general, any person would like to have more and more knowledge and this is especially true when it comes to health issues (5_52)  Q: Now, in the United States, if you became sick, where would you go?  A: So actually I don’t know, I swear to God, I don’t know. I know I was sent some insurance cards and also the organization set me up for some doctors appointments and even wrote the names of the doctors on those cards. But honestly, I don’t even know if those doctors are here in the hospital or somewhere else. I asked to have a doctor who is close to where I live. But again, when it comes to health and health issues, I become very confused and I don’t want to pay any attention for that, because my main concern is to find a job. But otherwise, if I die, that’s okay, I don’t have any problem (7_54)  Q**: Do you know what health insurance is?**  **A: Honestly, I don’t know. Actually I was told by one of my friends that I’m only gonna have this insurance for eight months and after those eight months if I didn’t get the renewal then I’m gonna have to pay for my medical issues out of my pocket, and that’s just gonna be so, so expensive (7_54)**  Q: And do you have any questions about healthcare, sorry – health insurance, anything you don’t understand that you’d like to know more about?  A: Yes. Very important to know the health insurance coverage, what does it cover and what it doesn’t cover is one question. And the other question, when I get – or anyone of family, is it the only way or the easiest way to make appointment online, or is there another way or easiest way (8)  Q: But who would you ask about health insurance coverage?  A: I don’t have any idea. Maybe I’d have to visit their website online. Is the only way I know (8)  Q: As a refugee and new citizen, what other health resources are available to you?  A: I have no idea (8)  Q: what information do you wish you had about the US healthcare system that you don’t have?  A: I want to know general information. When in the future I have question or inquiry I can go to the website, at least I have a background about the system. So general information is good for me including How the system works. Why am I covered in some fields and other fields are not covered? I’m just not – this information, I want to know that. (8)  Q: And what are some things about healthcare in the US that you found surprising or that you weren’t expecting?  A: The technology maybe. I told you before that [inaudible 00:12:28] online and follow up with doctors online, I think is very good, very good healthcare in United States. But it’s very difficult to get medication, because in Iraq it’s very different way to get medication. You can just go to the pharmacy and you can get any medication. But here, it must be a prescription. I mean in Iraq, some medication you cannot get, specific medications. But about 90 percent you can get [inaudible 00:13:05] at pharmacy. It’s different here and we need to understand how to get this medication. To me, I’m a refugee, come here, needs to understand this procedure, because it’s different (8)  Q: Is there anyone in your life who helps you take care of your health?  A: No one. (9_55)  Q: Is there anyone in particular that you go to or ask if you have – if you need advice or questions about your health?  A: No one. (9_55)  Q: When you have questions about your health, who do you ask?  A: No one. (9_55)  Q: And now in the US, if you became sick, where would you go?  A: Even here, I know that if I fall sick, I need to see the doctor. But because I don’t know if I have the means to. But then I was received by the [inaudible 00:15:16] and they told me that they would help me for three months. One month is already over, so I’m in the two months now. So when I get sick, I tell them. But back home, in Congo [inaudible 00:15:36] help take me to see a doctor (9_55)  Q: So the agency told you you would have insurance for 3 months?  A: Yes. They told me that I will – that after three months they will stop the health and then I’ll have to take care of myself. I’ll have to find a job and take care of myself (9_55)  Q Did the agency tell you about any health resources that will be available to you  A: No (9_55)  Q: Besides the agency that told you some of this information, is there anyone else who helped you or talked to you about healthcare resources?  A: I don’t know anyone (9_55)  Q: And who’s advice do you trust for taking care of your health?  A: Doctor’s. Everybody that knows about health (10)  Q: So if you have questions about your health, who do you ask?  A: Okay. I have no – I don’t have a way of knowing anybody here. I’ve been over here for about 35 days now. I do not know anybody. So the answer is no, nobody (11_57)  Q: When you have questions about your health, where do you go?  A: You’re off topic sir. **I’m sorry Ma’am, I had to – he just went two ways because he was [inaudible 00:05:43]. He said, well, ten days ago I felt that my sugar level was down, so I went to the dentist. It was the people at the dentist office who measured my sugar level and then they referred me to the hospital. I don’t have a machine to measure my sugar level. I’m not working. Yes, I have medications, but I’m not sure if I’m doing well with them or not. And for the nutrition, like I said earlier, the food here has hormones and [inaudible 00:06:07] and stuff, and it’s bad for me (11_57)**  Q: Now, in the United States, where would you go if you were sick?  A: I do not know, because I’m new here in the United States, I do not know. I only know one thing.  **If my condition worsens a lot, then I can just dial 911**. Other than that, no information at all (11_57)  Q: And do you know how long your insurance will last?  A: They gave me that information. But because of my diabetes, I have been forgetful (11_57)  Q: Who do you ask when you have questions about healthcare?  A: Because I do not understand English, I ask whoever around me, if I have a question, I ask them. (12_77)  Q: Now in the US, where would you go if you became sick  A: The hospital (13_45)  Q: do you know where the hospitals are close to your house?  A: no  Q: How would you figure out how to get there?  A: Yeah well I request for an address. I mean it depending on what I’m – what the – this problem I have. So I would always get an address or I would request for an address where that hospital is (13_51)  Q: What do you know about health insurance?  A**: Well there’s a paper – okay I have a problem with the – getting the understanding what insurance is because I don’t speak English. But there is a paper they gave us that you can always use in case you go to buy medicine or that – is it expensive and all that**. It helps you to minimize, but it was for my child. So as far as me is concerned they haven’t given me anything like that. (13_71)  Q: Do you have any questions about anything we talked about  A: Well I will clarify this ma’am he is asking me about the health insurance and before you explain that a little bit I want to clarify this. So because he didn’t understand he seems not to understand Swahili very well. And so I tried to explain it in Espand. Or I tried to explain it in detail so he understand exact what I’m saying. So I tried to explain about the health insurance and say the health insurance would help you probably minimize your cost of medicine. For example if you go to buy medicine and you have health insurance instead of buying at a certain price it might be cheaper because you have health insurance. So when I explained that he’s trying to ask now how can he get that health – how can he get the health insurance? (13_103)  Q: Is there anyone who you trust to ask medical questions to  A: No, I don’t have anyone to ask (14_26)  Do you know any hospitals close to your house?  A: : I don’t know any hospital close to me, ma’am (14_35)  Q: how would you get there?  A: I don’t know what to do, ma’am – I’m not gonna lie to you. I just got here two months ago (14_37)  Q |
| Knowledge, Understanding & Perception of US healthcare system | Facilitators | Q: Who helps you to take care of your health? (1_47 line 69)  A: I’ve undergone – I’ve seen the doctor twice in the – in two appointments, I’ve seen the doctors, and they make the necessary checkups and things like that so – and they said I’m fine. So – but later on down the road, they will find me some – refer to some providers in my area, and so I will contact them if I need anything. But that’s it, so I don’t need to worry about my health very much (1_47 line 70-74)  Q: Other than doctors, are there any other places that you get information about health and health care, now or in the past? (1_47 line 93-95)  A: So before we came to the United States, we had to attend some kind of training programs, like – or classes, this and that all the time. So – and then – in doing those training sessions, we were taught about how to take care of our health once we are in the United States. And then, like the various resources that are out there when you need help with your health or how to keep a healthy lifestyle, things – we had to learn a lot of those things, so they – with those kind of education information, they either gave us brochures or they let us watch the – like the TV show or whatever about that (1_47 line 100-106)  Q; How do you like to learn information about your health  A: I would prefer to read actually, to get information about my health (2_48)  Q: And where would you get information to read about it  A: I just surf the net (2_48)  Q: Is there anything in the US that’s different than what you expected  A: Well there is too many things that I didn’t expect to happen, like the insurance for me and my wife, the agency will help us paying for our expenses for the first three months. There is the food card that is also a kind of benefit. We are buying food with it. And also the cash assistance. So I didn’t know that they exist. (3_49)  Q: And what are the things about being here that make you feel normal? What makes you feel happy when you’re here?  A: Yes. The first thing, oh this make me feel good, it’s that over here they care about my kid’s health, they care about the vaccines. They care about taken care of. They care about their education. The first thing they did, they put them in school, they start teaching them English and following up with them. It’s like one – one-to-one, care, which I really admire (4_51)  Q: Who do you talk to about your health and who do you get your information from about how to stay healthy?  **A: There is one person that if we need anything, anything that has to do with our health or anything, we go to him. He’s in an organization. He gives us the schedule to go to the doctor if we need to or something. But I guess that’s the person I can go to if I need anything, appointments with doctors or something or any advice. Other than that, when we came over here to United States, they check me out. I went to the doctors and everything and everything was fine. I think if something was wrong they would have just told me by now (4_51)**  Q: Are the other people that you get information about healthcare from other than doctors?  A: Yeah. Someone needs some advice of something, I just call the organization, because I still don’t know a lot of things here. So I go to them. They just tell me what to do. Plus, my husband can speak English very well. He was an English doctor – I’m sorry, an English teacher, I’m so sorry – where we lived, we lived at before. So we don’t have no problems. He can ask the questions and he can take care of the problem if there is any. (4_51)  Q: how did you feel when you learned that you were coming to the United States?  A: Yes. Actually I was really happy. And I think of that – when I heard we was coming to United States, that this is a blessing from God. This is proof that he is happy with us. And maybe he will continue and heal my child (5_52)  Q: What made you excited to think about coming here?  A: Well I was really optimistic about this, when someone knows he’s going to the United States with all the healthcare that’s being provided over there, it’s different. So that’s why we were so happy (5_52)  Q: And who do you get your information about health from, besides – other than doctors?  A: Only the doctors (5_52)  Q: What are some things that you wish your doctors knew about you, but you haven’t been able to talk about?  A: No, actually, I’ve said everything. There’s nothing that I wish for them to know that I didn’t speak about. They know everything about us (5_52).  Q: If you have any questions about health or healthcare, how do you learn about – how do you find answers to those questions  A: Yeah. Mostly I search the internet (7_54)  Q: And if you ever have questions about your health, who do you ask usually?  A: Well, I registered online for medicine. . To follow my health status and my family health status too. When I need a doctor I will make an appointment online. So I think it’s easy way to keep in touch with the hospital or the doctor (8)  Q: What do you understand about health insurance?  A: The agency – the HIAS agency for refugees taken care of health insurance. So I’ve just been told that for the children, Keystone First, the [inaudible 00:07:59] and Keystone First, and for the adults, United Healthcare. Actually I’ve not experienced the health insurance, but they are doing good, the agency (HIAS). (8)  Q: And now in the US, if you became sick, where would you go?  A: Even here, I know that if I fall sick, I need to see the doctor. But because I don’t know if I have the means to. But then I was received by the [inaudible 00:15:16] and they told me that they would help me for three months. One month is already over, so I’m in the two months now. So when I get sick, I tell them. But back home, in Congo [inaudible 00:15:36] help take me to see a doctor (9_55)  Q: So what are some things that make it difficult to take care of your health?  A: I don’t see that there is anything here, because I have a card and things like that (10)  Q: what kind of card  A: An access card (10)  Q; what does your access card do for you, what does that give you?  A: I can go to the pharmacy with that to get medication. I can pay for food with it (10)  Q: So how did you learn how to go to the pharmacy, to pick up medicines or to come here to the doctor?  A: Oh, okay. Yes, I understand now. Yes. There is an agent from the HIAS that helps us with all of that (10)  Q: Do you know which hospital you would go to or how you would get there?  A: Normally, we just tell HIAS and they send us agents that are in charge of guiding us (10)  Q: Did anyone, when you first arrived, talk to you about healthcare services or resources available to you?  A: Well, this organization, I think it’s called HIAS, they gave us a glimpse, if you will, about such information (11_57)  Q: And what did they [HIAS] tell you about resources available to you?  A: They told us that if anything happens, call the ambulance. They told us where the hospitals are. They told us where the pediatric doctors were, so on (11_57)  Q: When you first came here did anyone – including the agency that helped you – talk to you about any healthcare services or resources that you have access to  A: Well I was told that if I have any problem with the illness I can go to the doctor and if it’s far I can always go to the pharmist and they should be able to assist me and I can buy medicine from there (13_56)  Q: When you first arrived here, did you – did the organization that helped you did they tell you anything about healthcare or any of the resources or services available to you as a refugee  A: Yes, they help with the food stamps and with the health coverage – health insurance and they help me for the rent for few months. And they just – give us a check lately – last month (14_44)  Q: When you have questions about your health, is there anyone you ask or any certain place that you go to, to ask these questions  A: The doctor (15_17)  Q: If you have any questions, is there anyone in the agency that you would talk to  A: Everybody over there can help out (16_48) |
| Knowledge, Understanding & Perception of US healthcare system | Informal Social Network | Q: what would you do if you became sick when you’re in the United States (1_47 line 121)  **A: Now that I’m in the United States, I could go to any drug store or – and get a friend who speaks some English to buy – assist me to buy things like cough medicine or whatever (1_47 line 122-123)**  Q: And who do you usually call when you need help with things like that, which friends (1_47 line 124 -125)  A: friends that live in my neighborhood or friends who are also roomates (1_47 line 126)  Q; The friends who you ask for help during these times, how do they help you (1_47 line 127-129)  A: So say for like a minor illness like a cough, I would ask a friend, okay – say, hey guys, please come along with me to the store to help me buy some cough medicine and let the storekeeper know what the name is or what medicine we need – like that. But in the long run, who would help me every time? So I would just try to do it on my own maybe down the road (1_47 line 130-133  Q: If you have any questions about health care, who would you ask?  A: My neighbor or anyone that I have, lived here more than me (3_49)  Q: Who do you talk to about your health and who do you get your information from about how to stay healthy?  A: There is one person that if we need anything, anything that has to do with our health or anything, we go to him. He’s in an organization. He gives us the schedule to go to the doctor if we need to or something. But I guess that’s the person I can go to if I need anything, appointments with doctors or something or any advice. Other than that, when we came over here to United States, they check me out. I went to the doctors and everything and everything was fine. I think if something was wrong they would have just told me by now (4_51)  Q: Are there any people that help you with your health?  A: No. I don’t have nobody. Yeah, I don’t have any friends, I don’t have any family, family members over here. So just the end of this month gonna be six months since we’ve been here. So it’s so new. (4_51)  Q: Are the other people that you get information about healthcare from other than doctors?  A: Yeah. Someone needs some advice of something, I just call the organization, because I still don’t know a lot of things here. So I go to them. They just tell me what to do. Plus, my husband can speak English very well. He was an English doctor – I’m sorry, an English teacher, I’m so sorry – where we lived, we lived at before. So we don’t have no problems. He can ask the questions and he can take care of the problem if there is any. (4_51)  A: Yes. I am learning slowly. I am getting some idea from my friends. They told me that I should not have to worry about anything else, I should not have to be scared about anything else. Even [inaudible 00:19:40] should not have to worry about my kids. They will work by themselves. And everything will be fine, I can walk freely outside. So everything looks like very good (6_53)  Q: If you have a question about your health, is there a way that you like to learn about health information? Do you like to read it or talk to somebody about it or look on the Internet?  A: I like that, but I don’t know how to use Internet. There is one old guy, his name is Groom, so if I have any question, I will go to him and he will tell me about everything. He is a good guy (6_53)  Q: Is there anyone in particular whose advice you trust when you have questions about your health  A: So actually, no, there is no specific one. Actually my health is very good. It’s only the insomnia. And back in Egypt I didn’t have this problem, because in Egypt you can just survive a month or two without having money to pay for your rent, you can just borrow that much money, the thousand pounds, from a [inaudible 00:08:31] friend or from an Egyptian friend and it’s just easy. Here, in the states, it’s much, much more difficult. Because no one is helping, and actually work is that not easy and usually when you find a job it’s far and you get paid very little. So that’s what makes me feel very [inaudible 00:09:00] and I have that insomnia (7_54)  Q: Is there anyone in particular who you go to with questions about your health?  A: Actually I go to my brother. But he’s not here, he’s in Germany (7_54)  Q: And how does your brother help you when you have questions?  R: So actually yes, when I have questions I just go to my brother, actually he’s my older brother and he’s very educated person and he has a university degree and he knows life better. So actually yeah I just go to him whenever I have questions (7_54)  Q: Do you know what health insurance is?  A: Honestly, I don’t know. Actually I was told by one of my friends that I’m only gonna have this insurance for eight months and after those eight months if I didn’t get the renewal then I’m gonna have to pay for my medical issues out of my pocket, and that’s just gonna be so, so expensive (7_54)  Q: What are some challenges that you experienced when you got to the United States, over the past few months?  A: So actually I’ve been here for a month and ten days, that’s like 40 days. And probably the English language was one of the challenges. But actually I have – I can just deal with different people, I can just talk to different people. I have a very social personality. And again, my main challenge is work, as you know. I would say I know Syrian people who came with me on the same airplane. And they went to a different state, and when they call me, they said like they live in paradise. The benefits they’re getting from their state, where they have [inaudible 00:30:42] the rent, they offered them good jobs. And that’s why I’m just gonna wait for a month or two, if things haven’t changed, then I’m gonna probably have to move to a different state (7_54)  Q: And besides doctors, other than doctors, are there people that you ask when you have questions about your health  A: Yes, I have – there are some doctors in my family too, in Iraq. So I frequently ask them if – me or my family – when facing any issue. So I ask them too. (8)  Q: So you mentioned you registered for Penn Medicine online. How did you learn about that?  A: From my cousin. He’s here in United States for about maybe – less than one and a half year. So he’s experienced in all this stuff. He advised me about a lot of thing, talked to me about a lot of things to do, to keep in touch in many fields and location I go, like Penn Medicine [inaudible 00:06:36] bills, everything online. So I use this. (8)  Q: Is there anything else your cousin talked to you about with regard to healthcare?  A: Yes. He taught me also to find a dentist. And how to drop off the prescription, pick up the medication from the pharmacy and how to go to the pharmacy or to register, like registration for a specific pharmacy to go pick up a medication form. A lot of things. (8)  Q: So in a few months, if you became sick, where would you go, to get care?  A: Okay. I’m still go back to them and ask them for help, because they are the only ones that I know [HIAS]. So I’ll still go and ask them how I can go about it and how I can manage to see a doctor. And if my situation gets worse, then I can call 911 (9_55)  Q: Is there anyone else, maybe a friend or a neighbor who helps you? Anyone besides the people from HIAS? Or family?  A: Yes. I have a friend from Congo. We walk together. We go to the pharmacy and he also helps me to see the city, because he’s been here for three months (10)  Q: Who do you have when you have questions about healthcare?  A: I’ll ask my friends (12_80)  Q: So did you talk to anyone about health insurance – sorry – about healthcare in the United States before coming to the United States  A: Well I spoke to my parents when they were here. I was back home and they used to tell me that as far as the health system here it’s very good. And also the living standard is good as well. (13_87)  Q: how would you get to the hospital if you got sick?  A: If I get sick, then I will tell me friends and family to take me to the hospital (15_41)  Q: And do you think they would know where to go?  A: Yeah. Old friends who live over here for a longer time, they know where to go (15_43)  Q: so you have some friends who have been here for a while?  A: I have my sister (15_45)  Q: And does she help you with any questions you have?  A: yes (15_47)  Q: so it sounds like – so you would call her if you had any questions if you got sick, and she would maybe help you  A: yes (15_50)  Q: Who told you about the healthcare system in the US? A: When I used to talk with my friend, they used to say I have an appointment today with the doctor. I have an appointment with the doctor. That’s how I knew by myself (15_91)  Q: . Is there anything that you wish somebody would help you with or would have told you about health– about your health or healthcare  A: my cousin helps me with this (16_66) |
| Knowledge, Understanding & Perception of US healthcare system | Provider Relationship | Q: Who’s advice do you trust about taking care of your health? (1_47 line 75)  A: Now – right now, I am taking care of my health by – on my – with my own will. And then I follow the doctor’s orders, and the doctors get guidelines to – I don’t need to trust any other person for my health (1_47 line 76-78)  Q: And what do you wish your health care providers knew about you (1_47 line 162-163)  A: the doctor or the provider has to look through my health records or my health history. Other than that, I wouldn’t know my health issue (1_47 line 164-165)  Q: How do you normally get information about your health if you have questions  A: I go see a doctor  Q: Is there anything you wish your doctor knew about your health, that they don’t know?  A: No, there’s nothing actually new to tell my doctor (2_48)  Q: Is there anything else, in particular, that you wish you knew – that your physicians knew about you or your family?  R: No. This is my life and I’m okay for the doctor to know everything about me (3_49)  Q: what do you wish that your healthcare providers or doctors knew about you?  A: Yeah, I told the doctors everything I know about my health. I didn’t hide anything since day one and now too, everything’s on the computer I guess, on the system. (4_51)c  Q: And who do you get your information about health from, besides – other than doctors?  A: Only the doctors (5_52)  Q: What are the things that you wish that they knew that maybe you haven’t talked to them about?  A: Oh no, nothing (10)  Q: Is there anything that you wish your doctor knew about you, that they don’t know?  A: No, no. I have explained everything. And everything I said was recorded in the computer, if you will (11_57)  Q: What were some things that made your experience coming to the US easier?  A: Okay. So speaking about going to the doctor or going to the hospital, it is much better than before. It even makes you feel psychologically better and comfortable, because they treating you better here (11_57)  Q: whose advice do you trust for taking care of your health?  A: doctors (12_20)  Q: If you have questions about your health, who do you ask?  A: I ask the doctor (14_24) |
| Access to US healthcare | Pre US | Q: before coming to the US, what would you do when you became sick (1_47 line 107))  A: I would visit the health clinics (1_47 line 108)  A: When we were in Malaysia, there were – I forgot to tell you that when we were in Malaysia, we had little [beeping] [inaudible 00:18:31] was run by Burmese people, and we could ask and buy a little – like little over the counter – what you would call over-the-counter medicine here. So we could get them at the Burmese stores, and then we would buy them and take them like over here. We would, some for a small major – a small – minor illnesses, we would take the over-the-counter pills. But for anything that’s more than – not too minor, we would just go to the medical clinic (1_47 line 111-117)  Q: Before coming to the United States, when you got sick, where would you go  A: Usually actually as a kid, when I got sick my parents took me to hospital, to the doctor (2_48)  Q: Back when you were in Syria, if you got sick, where would you go to get help?  A: Well, that would be to the doctor if I would be getting sick or something and I need medical attention I would have to go to the doctor, even though I financially, it’s not something that we can all afford. But you can say I can go just for the..inaudible (3_49)  Q: Tell me just a little bit more about what you’re experiencing [with regards to your health].  A: Well, I was back in Nepal when my feet were swelling, they said it was because of the sun. And when it is swelling, they sometimes told me that it is because of cold. But they don’t like to treat me over there, UNFCR also doesn’t like to treat me. Now I came here. Now I hope that my sickness will come out and everything will be checked and find out the diagnosis, what is wrong in me and they will find out individually what is going on inside (6_53)  Q: can you tell me how that makes you feel [not getting proper treatment in the past]?  A: I feel that when I was alone I don’t feel any sleepy at all at night, so I feel sleepy during the daytime. But in Nepal they think that it is bad to sleep during the day. I feel to sleep outside, but it is not good in our culture that people will – they will not feel good if I sleep outside. So I went to the doctor and they gave me sleep pills. And that pill helped me to sleep and I sleep in a good way to seven months. And after seven months again, my kids they grew up and when they were not at home, I started feeling the same thing, no sleep at night. When the kids are at home, then I feel comfortable and I sleep. When the kids are outside, I don’t feel any sleepy. So in this way I spend my days. (6_53)  Q: Back when you were living in Nepal, if you got sick, where would you go?  A: I use to go to hospital. Where should I go (6_53)  Q:When you were in Syria, where would you go when you became sick  A: So actually I use to see at the clinic. In Syria we have the public hospitals which are too, too, bad, and the private hospitals which are very expensive. So I use to see doctors and clinics. And actually I have two doctors who are doctors and I use to see them whenever there was something that was not urgent (7_54)  Q: Okay. So when you were in Iraq, where would you go when you became sick?  A: Doctor. Special clinics. Or to hospital. (8)  Q: when you were living in the Congo, where would you go if you became sick?  A: I would go to the doctor (9_55)  Q: And when you were in Togo, where would you go when you became sick?  A: In Togo, we go to the hospital. We had a card like this, the access card. And this card covered 80 percent of the fees and we were responsible for 20 percent (10)  Q: Back when you were in Egypt, where would you go when you got sick?  A: In Egypt, it’s very easy to find a doctor. You just go to the clinic or the hospital and you can find them and meet them easily (11_57)  Q: And how would they help you there in the clinic or hospital?  A: It’s a little bit of suffering, because the level of medicine as a science, is not good (11_57)  Q: How about back in Syria, where would you go if you got sick?  A: Same answer. Whenever you get sick, you just go to the clinic, if you cannot find a doctor there, then you go to the hospital. They receive you (11_57)  Q: when you were in Malaysia, where did you go when you becamse ill?  A: Clinic (12_24)  Q: when you were living in the Congo, where would you go if you became sick?  A: : I used to go the pharmacist. I used to go to pharmacies, small hospitals that used to be around us. (13_42)  Q: When you were back in Sudan where would you go if you became sick  A: Yes, it’s like – because I’m far away from the hospital or anything from a village in Sudan if I get sick or something I have to go all the way to see a doctor on the nearest city close to us (14_29)  Q: when you were living in Nepal, where would you go if you became sick?  A: Health center, a hospital  Q: What would they help you with?  A: Okay, just like a clinic health post, like a [inaudible 00:04:31] clinic, and I used to go over there and get things to check. And if it’s a bigger problem, they used to, only for us to [inaudible 4:44] the doctor, [inaudible 4:45] the hospital (15_29)  Q: When you were living in Sudan, where would you go if you became sick?  A: The hospital. the doctors see me, give me medication or something – whatever I need (16_27) |
| Access to US healthcare | Post US | Q: Who helps you to take care of your health? (1_47 line 69)  A: I’ve undergone – I’ve seen the doctor twice in the – in two appointments, I’ve seen the doctors, and they make the necessary checkups and things like that so – and they said I’m fine. So – but later on down the road, they will find me some – refer to some providers in my area, and so I will contact them if I need anything. But that’s it, so I don’t need to worry about my health very much (1_47 line 70-74)  Q: what would you do if you became sick when you’re in the United States (1_47 line 121)  A: Now that I’m in the United States, I could go to any drug store or – and get a friend who speaks some English to buy – assist me to buy things like cough medicine or whatever (1_47 line 122-123)  Q: If you were to get sick now, for example, tomorrow, where would you go  A: I go to the clinic, you just introduced me  Q: Before giving you that information, where would you have gone, if you didn’t have those resources  A: I went straight to the hospital (2_48)  Q: Did you talk to anybody, prior to coming to the United States, about what your experience would be like?  A: So I didn’t speak to anyone, because the story when I was in Syria and Mohammad got diagnosed with the cancer and they told me that he needs a bone marrow transplant and he should reach out either to Turkey or to Jordan. And then I went to Turkey to try to treat him there. And they treated him, but they didn’t have transplant there. So after that I didn’t have any other options, just to apply for the United Nations to get a refugee to be in America and to try to treat him here. And I was updating his condition with this agency and they have all the papers from Mohammad. And even though after I took the approval for coming here, I still update the condition, all of the time (3_49).  Q: What might happen if you get sick  A: If it’s something simple, we take care of each other, my husband, me and the kids, we take care of each other. If it’s something easy and something we can take care of. But just like my husband, he got some problems with something [inaudible 00:22:43] lately, had to call 911 and we need to go to the hospital. And you know, the stuff we cannot take care of, we go tot eh doctors. That’s it. And we take care of each other. That’s it (4_51)  Q: You said your son has asthma and you called to make an appointment?  **A: Yes. I’m a little upset about something, because I called to get appointment for my son. He was not feeling good. He has asthma. And they told me they don’t have no appointment until June. I had to take him at 3:00 in the morning to the hospital. I called another time for my husband and another time I called for my daughter, she had a fever. I was hoping that they can be seen by their own doctor, but that did not happen. So I had to take them to the hospital. (4_51)**  Q: And do your husband and children, do they have a regular doctor who they see all the time?  A: Yes. My husband called the – yes, we do have a family doctor. And my husband called the number we got on the card for my children too. But at least they should have just given us the same day for a sick child, or next day, to be seen. But they didn’t. (4_51)  Q: Tell me about yourself  A: Well, my name is [inaudible 00:01:25] and I’m a mother for three. And since I came to the United States, we started taking our son, who has been diagnosed with blood cancer, to the hospital. And he’s been in the hospital since then. I switch places with his father. Sometimes the father stays with him and sometimes I stay with him, because we still have two daughters as well waiting for us at home. So when they need me, I go there, and the father will come stay with our son, and vice versa**. So this is our life now**. (5_52).  Q: So what are some things, that, when you’re talking to doctors or trying to schedule appointments, what are some things that make that easier for you?  A: When we first came here to United States and they discovered what’s going on with our son, they took him in immediately. And he didn’t leave the hospital since then. Tell you the truth, the doctors here are really nice. They give you proper time, and explain things and the way they deal with the person is really very nice way, I feel really comfortable with them. As for my doctor, her father, he is the one who is taking care of all the appointments and scheduling them. So mainly he’s the one who is dealing with this thing (5_52)  **Q: Now, in the United States, if you became sick, where would you go?**  **A: So actually I don’t know, I swear to God, I don’t know. I know I was sent some insurance cards and also the organization set me up for some doctors appointments and even wrote the names of the doctors on those cards. But honestly, I don’t even know if those doctors are here in the hospital or somewhere else. I asked to have a doctor who is close to where I live. But again, when it comes to health and health issues, I become very confused and I don’t want to pay any attention for that, because my main concern is to find a job**. But otherwise, if I die, that’s okay, I don’t have any problem (7_54)  Q: Is there anything that you wish your doctor knew about you, that they don’t know?  A: Oh yeah, because yeah – there is somebody else that interprets us. So I cannot even tell you. (7_54)  Q: Is there anything else your cousin talked to you about with regard to healthcare?  A: Yes. He taught me also to find a dentist. And how to drop off the prescription, pick up the medication from the pharmacy and how to go to the pharmacy or to register, like registration for a specific pharmacy to go pick up a medication form. A lot of things. (8)  Q: And what are some things about healthcare in the US that you found surprising or that you weren’t expecting?  A: The technology maybe. I told you before that [inaudible 00:12:28] online and follow up with doctors online, I think is very good, very good healthcare in United States. But it’s very difficult to get medication, because in Iraq it’s very different way to get medication. You can just go to the pharmacy and you can get any medication. But here, it must be a prescription. I mean in Iraq, some medication you cannot get, specific medications. But about 90 percent you can get [inaudible 00:13:05] at pharmacy. It’s different here and we need to understand how to get this medication. To me, I’m a refugee, come here, needs to understand this procedure, because it’s different (8)  Q: Is there anything that you don’t understand about that procedure now?  A: Actually I think it’s good because I have already went to that pharmacy, dropped that prescription and get medication for my children, multiple times (8)  Q: And what has made your experience as a refugee more difficult?  A: More difficult for refugee? Maybe the healthcare, because a refugee come here, doesn’t know how to get in the system, the healthcare. And the places. But now I’m familiar with the transportation so I have no problem. I have GPS and transportation. But everybody comes here for the first time, the roads, places, very complicated. So it’s very important to get GPS (8)  Q: what does your access card do for you, what does that give you?  A: I can go to the pharmacy with that to get medication. I can pay for food with it (10)  Q: now in the US, where would you go if you became sick?  A: The hospital (10)  Q: When you have questions about your health, where do you go?  A: You’re off topic sir. I’m sorry Ma’am, I had to – he just went two ways because he was [inaudible 00:05:43]. He said, well, ten days ago I felt that my sugar level was down, so I went to the dentist. It was the people at the dentist office who measured my sugar level and then they referred me to the hospital. I don’t have a machine to measure my sugar level. I’m not working. Yes, I have medications, but I’m not sure if I’m doing well with them or not. And for the nutrition, like I said earlier, the food here has hormones and [inaudible 00:06:07] and stuff, and it’s bad for me (11_57)  Q: Are there any questions you have about your health or about healthcare that you wish someone would help you with  **A: So what I said earlier, I went to the dentist, they examined my sugar level, they referred me to the hospital. I went to the hospital there. They did some bloodwork for me. But they did not tell me about the results. I would like to know about the results at least (11_57)**  Q: have they [your children] been able to go to the doctor?  R: Yes, they did (11_57)  Q: what about now, where would you go if you became sick?  A: the hospital (12_26)  Do you know any hospitals close to your house?  A: : I don’t know any hospital close to me, ma’am (14_35)  Q: how would you get there?  A: I don’t know what to do, ma’am – I’m not gonna lie to you. I just got here two months ago (14_37)  Q: is there a hospital near your house?  A: near my house, yes (15_35)  Q: Now in the US, where would you go if you becamse sick?  A: My family doctor (16_29)  Q: Where would the ambulance take you if you called?  A; There is a hospital close to my house (16_34) |
